# Multisensory sensitivity in relation to pain: a scoping review of terminology and assessment

**DOI:** 10.1097/PR9.0000000000001193

**Published:** 2024-10-25

**Authors:** Harper Dunne, Laura A. Frey-Law

**Affiliations:** Department of Physical Therapy and Rehabilitation Science, Carver College of Medicine, University of Iowa, Iowa City, IA, USA

**Keywords:** Multisensory sensitivity, Sensory sensitivity, Somatosensory amplification, Hypervigilance, Sensory defensiveness, Sensory over-responsiveness, Sensory processing disorder, Pain, Chronic pain, Survey, Questionnaire

## Abstract

Chronic pain is a debilitating health problem affecting 20 million Americans annually. Most patients with chronic pain report negative impacts on daily function and quality of life, which can result in devastating emotional and financial stress. Although the causes of chronic pain remain elusive, there is increasing interest in sensitivity to everyday sensory stimuli as it relates to chronic pain, potentially serving as an indirect marker of altered central nervous system sensory processing. However, sensitivity to multiple sensory inputs, eg, bright lights, certain fabrics, loud noises, etc, is described using multiple terminologies. The lack of a common vocabulary makes it difficult to find and summarize related discoveries, potentially inhibiting scientific progress. Thus, the purpose of this scoping review was to identify and characterize the terminology used in publications assessing some form of multisensory sensitivity as it relates to pain (eg, a pain cohort or pain sensitivity). Our review of 6 databases (PubMed, Scopus, Embase, CINAHL, PsycINFO+, and Cochrane) comprehensively cataloged peer-reviewed studies published through March 2023 in this domain. Of 12,841 possible studies identified, 92 met all inclusion criteria, with over 80% being published in the last decade. A wide range of terminology has been used for this construct, likely in part a result of the many different professional disciplines represented. These results provide valuable insights for future development of a standardized vocabulary and serve as a resource to aid future investigators of multisensory sensitivity and pain in their study design.

## Abstract 2

Supplemental Digital Content is Available in the Text.

No common terminology exists for sensitivity to multisensory stimuli as it relates to pain. This comprehensive scoping review summarizes the available literature for future researchers.

## 1. Introduction

Identifying biomarkers or risk factors of chronic pain are central themes of many investigations aiming to improve interventions for pain. Differentiation of peripheral vs central underlying pain mechanisms has garnered extensive debate with the ultimate goal of allowing for more targeted pain treatments.^14^ While central pain mechanisms are inherently challenging to identify in clinical populations, multiple techniques have been used as indirect measures for central sensitization, such as quantitative sensory testing (QST), functional magnetic resonance imaging, electrophysiological assessments, clinical phenotypes, and self-report surveys.^1,13,21,30,34,53^ Several authors have also suggested that generalized sensitivity to normal daily sensations might serve as a surrogate or indirect marker of generally facilitated central nervous system (CNS) sensory processing and, more specifically, central sensitization.^5,15,16,23,46,56^ These assertions are supported by the evidence from several neurophysiological studies demonstrating shared processing of painful and nonpainful stimuli in the CNS.^33,36,47^ Similarly, Bar-Shalita et al.^7^ have proposed a neurophysiological model linking altered sensory processing and pain processing. Thus, there is widespread agreement linking painful and nonpainful sensory inputs (See Table S1 in Supplemental Materials 1 for a complete list of abbreviations, http://links.lww.com/PR9/A252).

There is both a wide terminology used to refer to this self-reported sensory sensitivity and a variety of different assessments for this trait in the literature. For example, terminology ranges from relatively similar (eg, multisensory sensitivity [MSS] or generalized sensory sensitivity [GSS]) to those that seem to be related yet may have distinct qualities (eg, somatosensory amplification, sensory hypervigilance, or sensory over-responsiveness).^3,39,49,55,57^ There has been little attention more clearly devoted to either unify or differentiate between terms and survey instruments used. Since investigating sensory sensitivity is a relatively recent area of interest within the field of pain science, this heterogeneity of terminology and assessments complicates interpretation of results and serves as a barrier to effective communication between multidisciplinary researchers and clinicians. For example, the lack of consistent terminology limits effective comprehensive literature searches, impairs interpretation of methodologies and results between studies, and ultimately hinders scientific advancement in this area.^26,42^ Recognizing that several terms and instruments are used in the study of this general construct, for simplicity, we will use “MSS” as a representative term for this construct throughout this review. This is not intended to negate or minimize the potential for unique aspects within the overall umbrella of sensory sensitivity to be reflected through various terminologies and assessments used in the literature.

There are multiple instruments used to assess aspects of MSS reported in the literature. In a scoping review of sensory processing disorder assessments for autism spectrum disorder (ASD), where sensory abnormalities are diagnostic hallmarks of neurodivergent conditions,^10,19,22,26^ a range of methodologies were identified.^26^ Sensory abnormalities associated with ASD range from sensory hypersensitivity with or without sensory avoidant behaviors to sensory hyposensitivity and/or sensory seeking behaviors, as described by the Dunn model of sensory processing. While a range of testing paradigms was noted, including neuroimaging, direct observation, psychophysical testing, and qualitative methods, the most commonly used was a self-report or proxy survey approach.^26^ The advantage of self-report is that it is highly feasible for both clinical and research applications and provides a person-centered perspective. The tools used for ASD diagnosis may not fully overlap with those used for screening MSS in pain cohorts. Thus, given the growing interest in this area of pain research and the inherently multidisciplinary interest in sensory sensitivity, we identified a need for a comprehensive characterization of the terminology and assessments of MSS as it relates to pain available in the literature.

Thus, the purpose of this study was to perform a scoping review of studies involving self-report or proxy assessment of MSS, as it relates to pain in some way. Our primary goals were to identify and catalog the terminology and instruments used to represent aspects of the MSS construct. In addition, we aimed to summarize the rate of annual MSS-pain publications, the populations investigated, and the journals in which they were published. Our primary intention was to create a resource that summarizes the key features of existing literature in this field. This resource could potentially promote communication among clinicians and researchers from a variety of disciplines; a better understanding of the breadth of the evolving MSS-pain literature; consensus-building endeavors; and enhancement of both the clarity and precision of terminology. In these ways, we hope this resource may contribute to efficient scientific advancement, ultimately leading to improved targeted interventions for chronic pain.

## 2. Methods

Preferred Reporting Items for Systematic Reviews and Meta-Analysis extension for Scoping Reviews and Joanna Briggs Institute (formerly known as Joanna Briggs Institute) Scoping Review Network scoping review frameworks were followed in designing and implementing this study.^35,37,52^ The scoping review protocol was registered in Open Science Framework on April 27, 2021, “MSS Scoping Review,” with subsequent file uploads including search strings, review criteria, and standardized instructions for our review team. Two comprehensive literature searches were performed in 6 databases: PubMed, Embase, SCOPUS, Cochrane, PsycINFO+, and CINAHL (more details below). The first search was performed on November 24, 2021, and repeated on March 31, 2023, searching all available dates for each database (ie, no start date defined to encompass all available citations possible). Last, a third strategy identified additional articles published within our original timeline from the reviews of cited literature or other means (eg, reviewer suggestion) and if criteria were met, they were added to the database.

### 2.1. Literature search

Because of the nature of the research question (to identify the wide range of terminology and survey instruments used without assuming complete a priori knowledge), a search strategy was designed to be overly inclusive of possible relevant literature. To be included, studies must have met the following inclusion criteria: (1) published as a full-length, peer-reviewed article in an academic journal; (2) available in English; (3) included a means of assessing participants' self-report(s) of MSS (eg, operationally defined as 3 or more sensory domains; light, sound, touch, hearing, etc); and (4) involved pain in some way (eg, involved a pain population, assessed pain in a different clinical population, or assessed pain sensitivity in healthy adults). Exclusion criteria included as follows: a(1) “gray” literature (ie, dissertations, conference abstracts, conference proceedings), (2) studies involving only psychophysical sensory testing or imaging (eg, QST or functional magnetic resonance imaging), (3) studies that only assessed one or 2 sensory sensitivities (eg, photophobia or phonophobia only), (4) nonhuman studies; (5) studies unavailable in English, and (6) studies for which we could not retrieve full-text documents despite multifaceted efforts (including interlibrary loans). The inclusion of peer-reviewed publications only was chosen based on our goals of identifying MSS terminology in published literature.

We generated our search string as 2 blocks, one targeting the sensory sensitivity portion of our inclusion criteria and the other targeting the pain portion. These 2 blocks were combined using the “AND” Boolean operator to find studies, which investigated both pain and MSS in some manner. See Figure S1 in Supplemental Materials 1 (http://links.lww.com/PR9/A252) for a visual conceptualization of the search string methodology as well as the full search strategy used for the PubMed search. The search string was developed and reviewed by the 2 authors, with extensive assistance and consultation by a reference librarian experienced in scoping reviews.

The “MSS block” consisted of both a series of identified “core terms,” such as “sensitivity,” “hypervigilance,” and “over-responsiveness,” representing the notion of sensitivity or hypersensitivity, and “modifier terms,” which often clarified the nature of the sensitivity in some way (eg, “multi,” “generalized”) or specific sensory domains (eg, photophobia, phonophobia). Within each subset, all identified terms (ie, all “core terms” or all “modifier terms”) were each connected with the “OR” Boolean operator to be maximally inclusive. These 2 sub-blocks were then connected with the “AND” Boolean operator to better identify studies investigating sensitivities of multiple sensory domains (see Supplemental Materials 1, http://links.lww.com/PR9/A252). In addition, known MSS-related surveys were also included in the MSS block, each connected with the “OR” Boolean operator. The “pain block” was designed to include (1) all pain conditions listed on the American Chronic Pain Association website and (2) pain assessment terminology (eg, “chronic pain,” “hyperalgesia,” “sensitization,” etc), each connected with the “OR” Boolean operator to be maximally inclusive.

The search string was iteratively tested using 11 preidentified sentinel articles, with adaptations made as needed to ensure the final string successfully captured those known publications. The search string was first developed for PubMed and then translated to each of the other 5 databases following their respective controlled vocabularies.

### 2.2. Screening process

The citations identified from the 6 database searches were deduplicated following the multistage Bramer method.^11^ The resulting titles and abstracts were then screened by 5 individuals trained in the inclusion/exclusion criteria using Rayyan screening software, with a minimum of 2 reviewers per citation. Any reviewer conflicts were discussed and resolved collectively by the team. The full-text versions of citations which passed this initial screening phase were then pulled and screened by an assigned primary and secondary screener (of 4 trained individuals), considering all inclusion and exclusion criteria. Any conflicts were again resolved by group consensus.

The search process was repeated in March 2023 to find additional publications from the 15 months between the 2 searches. The second search retrieval and screening followed the same protocol as the first search with the addition of deduplication occurring between the first and second search results by database (ie, search time frame was not limited to the date of the first search to minimize the risk of missing publications that were cataloged after publication date).

### 2.3. Data extraction and analysis

If after full-text review, a study was deemed to meet the inclusion criteria, data were extracted by one of the trained research assistants, with subsequent data checking by a second to minimize recording errors. From each included study, the following information was recorded in Excel: title, author, publication year, journal, population(s) studied, MSS terminology, and MSS surveys used. As several known MSS phrases involve multiple terms (eg, MSS or somatosensory amplification), terminology was cataloged by “core” (eg, sensitivity, amplification, hypervigilance, over-responsiveness, etc) separate from “modifier” terms (eg, multisensory, sensory, somatosensory, etc). In addition, descriptions and definitions of the MSS construct as provided in the manuscripts by authors, if available, were extracted. When authors used more than one term to describe MSS (eg, one in their introduction, but another in the methods or discussion), we made our best effort to identify which was used as the “primary” MSS term for each article, with the review and consensus by the 2 authors. Self-report instruments used to assess MSS were identified, and efforts made to extract original descriptions of the construct from their source validation and reliability studies.

### 2.4. Analysis

Summary statistics (ie, frequencies) were computed for number of studies, journal counts, patient population(s) studied, MSS terminology, and survey methodology. The extracted MSS terminology was then separated into “core” and “modifier” components, and journals were categorized by field of study. The resulting frequencies of all key variables were summarized using Excel pivot tables and visually represented using GraphPad Prism. To estimate publications per year for 2023, the resulting number of identified studies up until the search date of March 31, 2023, (ie, for 1 quarter of the year) was annualized to 12 months. The qualitative thematic analysis focused on identifying common themes across studies used to define or describe the MSS construct, with minimal quantitative analysis. Given the increased interest in this topic in the last decade or so, in addition to summarizing study characteristics over all publications identified, we also separated the studies into 2 temporal categories: past decade (2014 and after) and pre-2014.

## 3. Results

### 3.1. Search results

**Figure 1.**
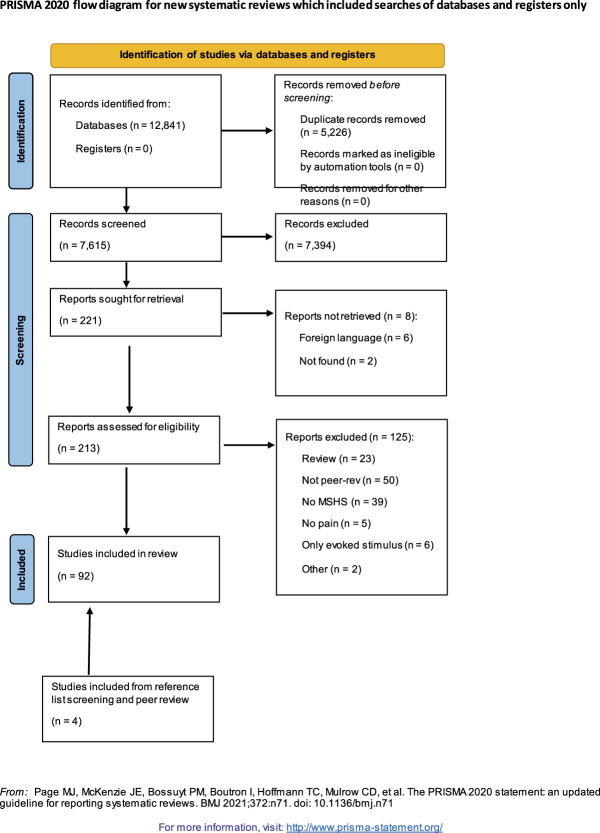
Preferred Reporting Items for Systematic Reviews and Meta-Analysis 2020 flow diagram of the search and screening results of this scoping review.

A total of 12,841 records were identified and 7,615 screened after deduplication from the comprehensive search of the 6 databases summed across both searches (see Fig. 1 for Preferred Reporting Items for Systematic Reviews and Meta-Analysis flow diagram). Of the 221 publications identified for full-text screening, 213 full texts were retrieved and reviewed (2 were unavailable despite exhaustive search; 6 were not available in English). From these, a total of 88 studies were identified as assessing self-reported MSS in either a pain population or relative to pain assessments. Four additional articles were identified from reviewing citations or other sources and thus included in this scoping review, for a total of 92 included articles. See Table S2 in Supplemental Materials 2 (http://links.lww.com/PR9/A252), for more details on each included study.

### 3.2. Publication timeline

**Figure 2.**
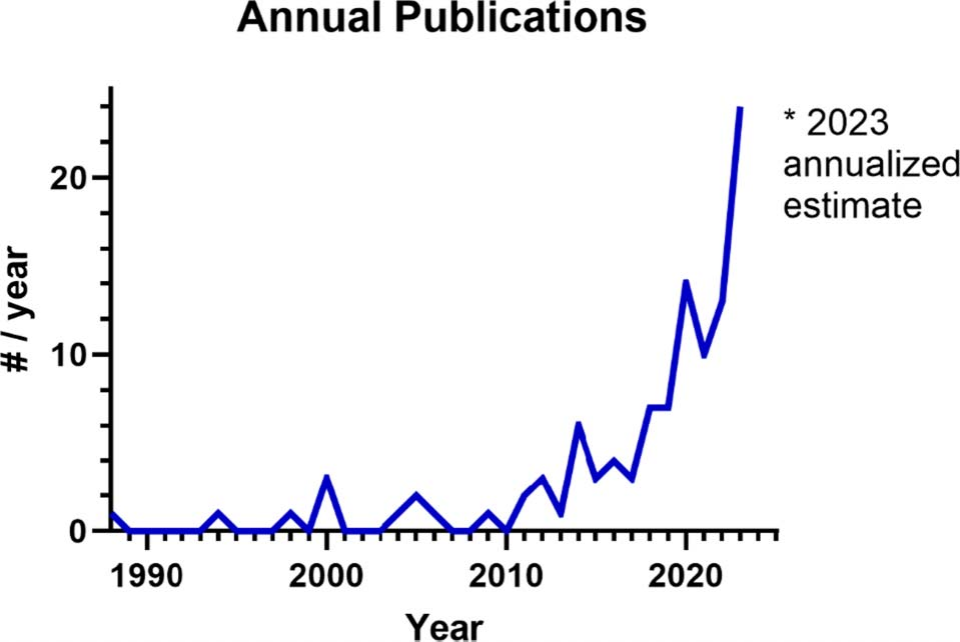
Publication count timeline as annualized number of MSS-pain publications per year. Note the first publication found was in 1988. For 2023, the number of publications was annualized based on 8 manuscripts found as of March 31, 2023. MSS, multisensory sensitivity.

The earliest MSS-pain publication identified was published in 1988. The annual rate of publication was relatively flat over the next 2 decades but increased substantially in the past decade (Fig. 2). In fact, 82% (75/92) of the MSS-pain studies found were published since 2014.

### 3.3. Multisensory sensitivity nomenclature

**Figure 3.**
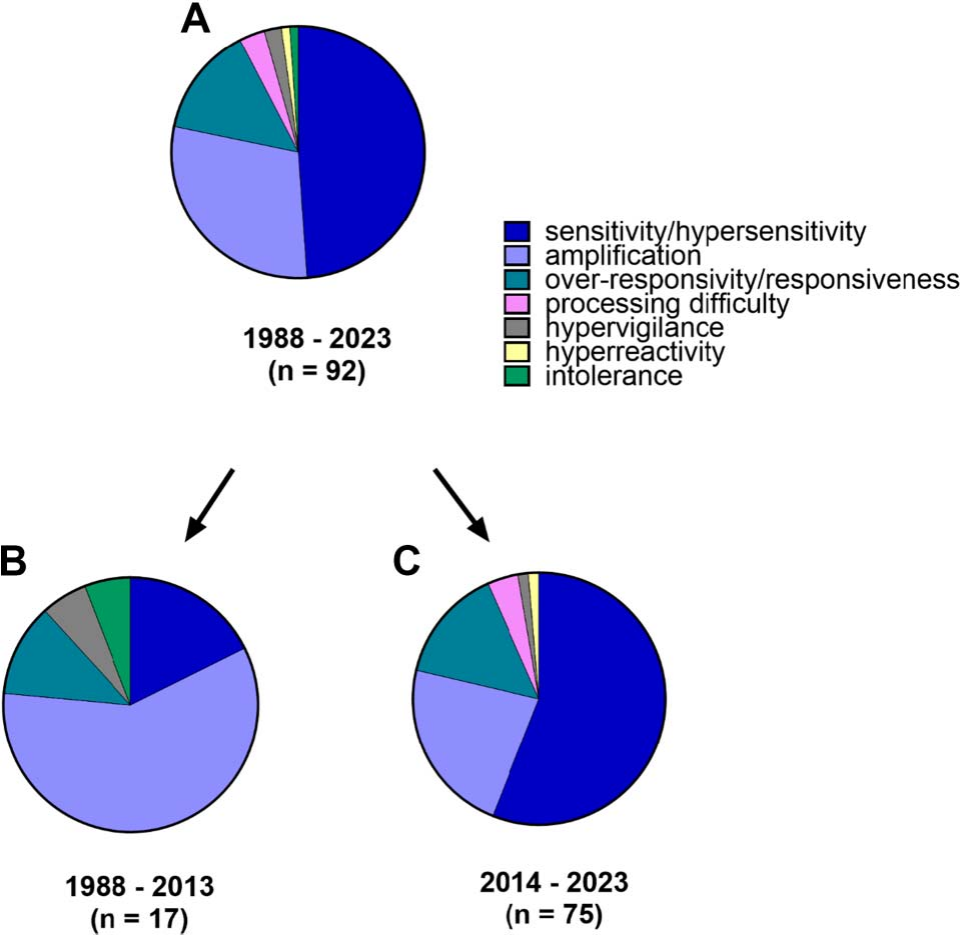
Proportion of “core” terms used to represent an MSS-related construct as it relates to pain: (A) in all studies (n = 92), (B) in studies published before 2014 (n = 17), and (C) studies published in the last decade (2014–2023) (n = 75). MSS, multisensory sensitivity.

**Figure 4.**
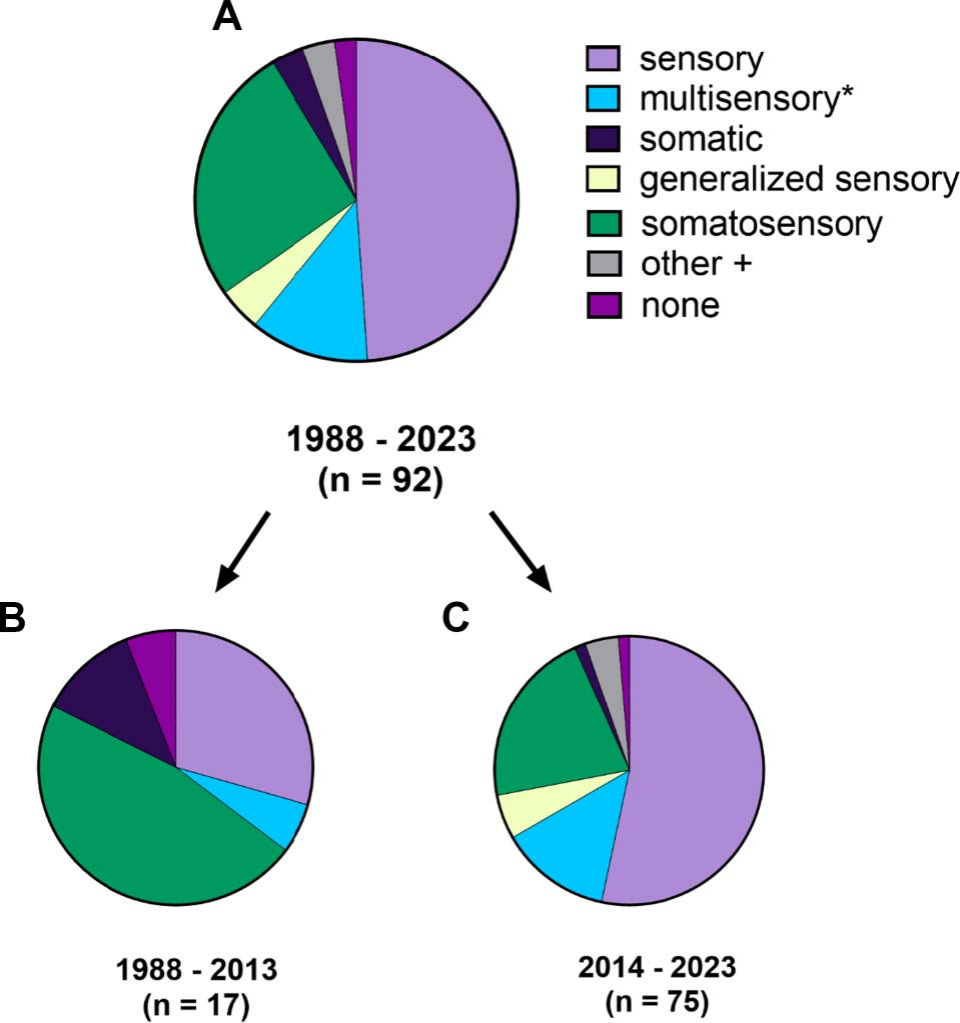
Proportion of “modifier” terms used to represent an MSS-related construct as it relates to pain: (A) in all studies (n = 92), (B) in studies published before 2014 (n = 17), and (C) in studies published in the last decade (2014–2023) (n = 75). *Multisensory includes 1 “multisystem sensory.” Other includes one of each of the following: “cross-modal,” “trait sensory,” and “interoceptive.” MSS, multisensory sensitivity.

In the 92 included studies, combinations of 7 “core” terms or phrases (Fig. 3) and 10 “modifier” terms or phrases were used to denote MSS-related constructs (Fig. 4). Three of the 7 core terms were each used in 10 or more studies: (1) sensitivity/hypersensitivity (n = 45), (2) amplification (n = 27), and (3) over-responsiveness/responsivity (n = 13). The remaining 4 core terms were each used only 1–3 times each: processing difficulty, hypervigilance, hyperreactivity, and intolerance. While sensitivity/hypersensitivity was most frequently used overall, this represents a shift in the past decade, whereas amplification was predominate core term before 2014.

The “modifier” terms most often denoted the involvement of the somatic and/or sensory system as well as the multinature, generalized nature, or trait nature of the sensitivity. The 3 most common modifiers included (1) sensory (n = 45), (2) somatosensory (n = 24), and (3) multisensory or multisystem sensory (n = 11). Multiple additional modifiers were each used in 4 or fewer studies: generalized, generalized sensory, somatic, cross-modal, trait sensory, interoceptive, and no-modifier. Before 2014, the most common modifier was somatosensory (47%, 8/17), which was often but not always paired with amplification, the most common core term from pre-2014. Whereas, after 2014, simply “sensory” was used in 53% (40/75) of the studies, although somatosensory remained the second most common at 21% (16/75).

### 3.4. Multisensory sensitivity assessment measures

Most studies used at least one validated instrument (82%, 75/92), either specifically for the assessment of MSS or a subset/subscale of an instrument. These included a total of 18 different self-reported measures (across a total of 82 instances as 3 studies assessed MSS with multiple measures, see Table 1). The 4 most common MSS assessments were (1) the Somatosensory Amplification Scale (SSAS, n = 26)^9^; (2) the Adolescent/Adult Sensory Profile (AASP, n = 17)^12,29,41^; (3) the Sensory Responsiveness Questionnaire—Intensity Scale (n = 11)^3^; and (4) items from the Complex Medical Symptoms Inventory (CMSI, n = 6).^58^ See Table 1 for more details on each instrument. The remaining 14 MSS measures were used infrequently (1–3 times each).

**Table 1:** Multisensory sensitivity self-report instruments used and frequency (N) in the 88 included studies, with citations reported for scale description and psychometric properties.

Self-report instrument	Measured construct description(s)	Studies (N)	Scale citation(s)	# Items	Item type
Somatosensory Amplification Scale (SSAS) SSAS modified from original Multisensory Amplification Scale (MSAS)	Tendency to experience more intense, noxious, and disturbing somatic sensations or sensitivity to bodily sensations Somatosensory amplification, amplification of multisensory sensations, or multisensory sensitivity Multisensory sensitivity	24 2 3	^8,9^ ^8^ ^55^	10 7 or 15 12	5-point Likert scale—frequency
Adolescent/Adult Sensory Profile (AASP) Short Sensory Profile (SSP) Short Sensory Profile 2 (SSP2) Sensory Profile (SP)	Dunn model of sensory Processing, responses to sensory events in daily life, responsiveness to sensory input, behavioral responses to sensory input Atypical responses to daily sensory stimuli, used for clinical diagnosis of sensory modulation disorder	17 2 1 1	^12,27–29,41^ ^28^	60 (15 for sensory sensitivity subscale) 38 34 125	5-point Likert scale—frequency
Sensory Responsiveness Questionnaire—Intensity Scale (SRQ-IS)	Identification of sensory modulation difficulties through patterns of behavioral responses to daily sensations	11	^3^	58	5-point Likert intensity
Complex Medical Symptoms Inventory (CMSI) subset/Generalized Sensory Sensitivity (GSS)	Generalized sensory sensitivity	6	^58^	Varies from 4 to 7	Yes/No items
Body Vigilance Scale (BVS)	Excessive attention toward sensory events, hypervigilance for somatosensory signals	2	^43^	4	0–10 or 0–100 scale
Sensory Hypersensitivity Scale (SHS)	Sensitivity to sensory stimuli, sensory hypersensitivity	2	^24^	25	5-point Likert scale—agreement
Sensory Perception Quotient (SPQ)	Sensory hyper-sensitivity and hypo-sensitivity across 5 modalities, sensory disturbance	2	^51^	Short: 35 Long: 92	5-point Likert scale—agreement
Highly Sensitive Person Scale (HSPS)	Temperament associated with sensitivity to emotional, physical, and social stimuli	3	^2,31^	27	7-point Likert intensity/agreement
Highly Sensitive Child Scale (HSCS)	Environmental sensitivity	1	^40^	12	7-point Likert intensity/agreement
Body Awareness Questionnaire (BAQ)	Attentiveness to nonemotive bodily processes	1	^48^	18	9-point Likert agreement
Adult Sensory Questionnaire (ASQ)	Sensory defensiveness	1	^38^	26	True/false
Chronic Pain Questions (CPQ) subset	Sensitivity to bright lights, loud noises, smells in past week	1	^18^	1 of 14 items	0–10 scale
Adult Autism Subthreshold Spectrum (AdAS) subset	Hyper-sensitivity and hypo-reactivity to sensory input	1	^22^	17 of 160 items	Yes/no items
Sensory Processing Scale Inventory (SPSI)—Sensory over-responsivity (SOR) subscale	Sensory over-responsiveness, exaggerated responses to one or more sensory stimuli not perceived as harmful or noxious	1	^44^	76 items	Parent-report yes/no

A few studies used more than 1 instrument; 10 used custom items that were not reported; 7 used a combination of tools assessing single sensory sensitivities (ie, photophobia, hyperacusis, etc); and 1 did not report their assessment method.

The 17 studies (18%) not included in the above cohort, either used a combination of multiple unidimensional self-report measures (eg, assessments of photophobia only, phonophobia only, etc) to reflect MSS (n = 7) or custom items designed by the study authors (n = 10). Rarely were details of these specific custom survey or interview items provided.

### 3.5. Populations studied

**Figure 5.**
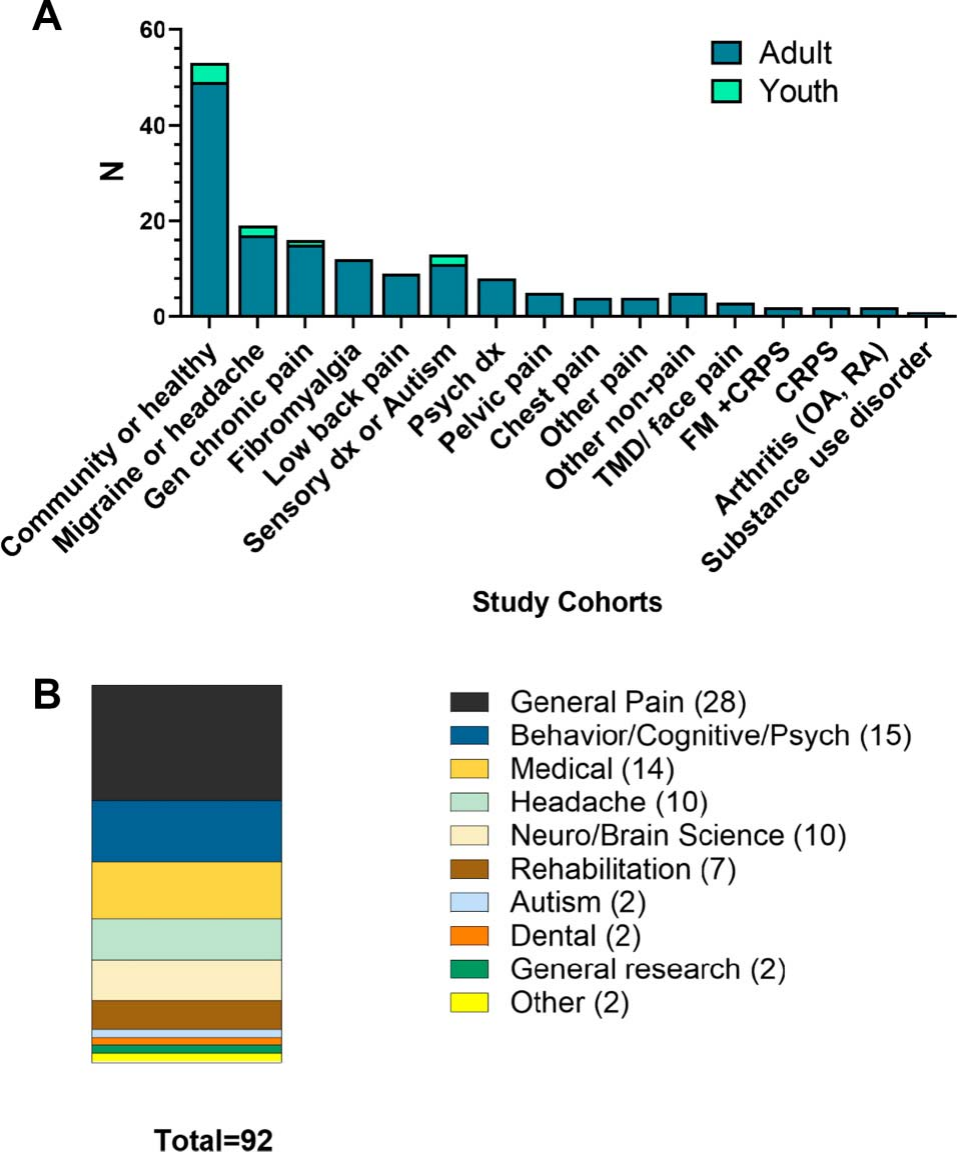
(A) The 157 cohorts included in the 92 studies (several included 2 or more cohorts) were almost exclusively performed in adult populations (dark bars) with only 6% in those younger than 18 years (light bars). The most common pain cohorts were migraine or headache, unspecified or mixed chronic pain populations, fibromyalgia, and low back pain. (B) The 92 MSS-pain studies were published across 68 unique journals, shown here as the distribution across 10 different journal disciplines. The most represented was general pain but followed by relatively similar distributions among psychological, medical, headache, and neuroscience journals. MSS, multisensory sensitivity.

Several studies (n = 49) included 2 or more populations; thus, the total number of populations identified (n = 157) exceeds the 92 included articles (Fig. 5A). Most (n = 78) included a clinical pain population; some involved a population, which was not based on pain status (n = 26, eg, psychiatric, sensory disorder, or general patient population); and 53 included a healthy or community-based cohort either as a comparative group or in studies of pain sensitivity or catastrophizing without a pain cohort. Of those with pain, the most common conditions included as follows: headache or migraine (n = 19), general chronic pain (n = 16), fibromyalgia (n = 14), and chronic low back pain (n = 9). For nonpain clinical populations, the most studied groups were those with a sensory disorder (n = 13) or psychiatric referral patients (n = 8). Pain assessments varied from reports of spontaneous pain or pain intensity to measures of pain sensitivity using QST or surveys. Nearly all cohorts were adults (n = 148/157, 94%), with only ∼6% involving pediatric cohorts. One study surveyed providers about their perceptions of this topic, thus was a unique cohort yet still met all inclusion criteria.

### 3.6. Journals

The 92 included studies were published in 66 different journals, representing a wide range of disciplines (Fig. 5B). The 2 most frequently represented journals were Pain (n = 8) and The Journal of Headache and Pain (n = 6). When divided into categories by discipline or theme, the 3 most common categories were general pain journals (n = 28), followed by behavior/cognitive/psychology journals (n = 15) and general medicine journals (n = 14).

### 3.7. Thematic multisensory sensitivity descriptions

Several themes were extracted from the MSS explanations and descriptions across studies (Table 2). These varied across 4 broad thematic categories: (1) the types of sensory stimuli involved or assessed, (2) descriptions of how sensitivity was conceptualized, (3) the MSS construct's relationship to pain, and (4) the impacts of MSS. Three types of sensory input were typically described, often with overlap: (1) external stimuli (light, sound, smell, touch, etc), (2) internal body sensations such as temperature or proprioception, and (3) visceral sensations such as hunger or heart rate. While few articles provided clear definitions of their chosen MSS term, the descriptions typically included a component of the perception of intensity, negative responses to one's senses such as bothersomeness, or the notion of over-responsiveness. While most studies targeted nonpainful sensations in their assessment of MSS, some explicitly included allodynia within the broader conceptualization of MSS. The fourth thematic category described by several authors included aspects of MSS's impact on patients' lives, such as anxiety, emotional distress, and generally reduced quality of life.

**Table 2:** Qualitative thematic analysis of multisensory sensitivity descriptions.

Theme	N (%) of studies	Theme subset	Theme definitions
Sensations Involved	41 (45%)	External stimuli	Involving sensitivity to external stimuli from the environment (eg, visual, auditory, olfactory, touch, or taste)
	17 (18%)	Somatic sensations	Involving sensitivity to temperature, vibration, and proprioception
	5 (5%)	Visceral sensations	Involving sensitivity to internal sensations related to organs within the torso, for example the heart, lungs, stomach, etc.
Characterizing or defining “sensitivity”	13 (14%)	Intensity—high	Involving the notion that the “volume dial” of a sense is turned higher than typical
	29 (32%)	Over-responsive	Involving over-responsiveness to or increased awareness of sensations with negative impacts (see below)
	24 (26%)	Bothersome/Disturbing	Involving sensory stimuli as interpreted as more bothersome or disturbing than typical
	3 (3%)	Under-responsiveness	Involving under-sensitivity or under-responsiveness to stimuli
Pain	26 (28%)	Noxious/allodynia	Involving sensitivity to such a degree that it becomes painful or noxious; and/or allodynia
	15 (16%)	Separate from pain	Involving sensory sensitivity that is distinct from pain (but perceived as related)
Impact	15 (16%)	Decreased quality of life	Involving generally reduced quality of life
	8 (9%)	Anxiety	Involving the psychological trait of anxiety
	8 (9%)	Emotional distress	Involving emotional distress

Themes identified and the frequency of articles (N, %) which included each theme in their descriptions of MSS.

MSS, multisensory sensitivity.

Based on these thematic analyses of MSS descriptions and our understanding of how the construct is described throughout the literature after reading all of the articles in detail, we generated a model of 2 common conceptual underpinnings involved in MSS (see Figure S2, Supplemental Materials 2, http://links.lww.com/PR9/A252). Generally, MSS conceptualization appears to mirror the biopsychosocial model of pain. For example, MSS descriptions and their associated assessment tools are preferentially described as either a psychological construct (eg, SSAS and its association with hypochondriasis) or as reflecting biological or physiological ramifications of CNS function. Within the overall category of biological/physiological underpinnings, various interacting factors from both the CNS, such as cortical hyperexcitability and sensory modulation alterations, likely play a role.^7^ Most descriptions, however, embody components of both the psychological and biological/physiological underpinnings. Although not as frequently linked to the conceptualization of the MSS construct, social aspects of MSS might be best represented by the impact theme, including anxiety and emotional distress. Some survey items or subscales that assess consequences of altered sensitivity, such as avoidance or seeking behaviors (eg, AASP sensory avoiding subscale), may also reflect social aspects of MSS.

## 4. Discussion

This scoping review catalogs the growth in publications and wide range of terminology used by researchers to represent the construct of MSS in the context of pain, highlighting the field's evolution. Some of the terms identified may not be immediately familiar or obvious to those interested in this area of research, although those terms might represent a construct that is either similar or the same to constructs the author is familiar with. Furthermore, this variability extends to several additional aspects of the MSS-pain literature, including the themes identified from the MSS descriptions provided in publications, the methodology used to assess MSS, the journal disciplines represented, and the populations studied. Accordingly, the results emphasize the importance of this review's purpose: compiling the existing literature to better characterize this evolving field and aid in future research efforts.

Despite the variability, the most notable commonality across publications was simply an acknowledgment of increased sensitivity to multiple sensory domains or, at the least, sensitivity that is nonspecific to only one domain. The most precise modifier terms to reflect this notion might arguably be the use of “multi’” or “generalized” with one of the core terms: sensory sensitivity, amplification, over-responsiveness, or hypervigilance. However, the use of “sensory sensitivity” in isolation, without additional specification, was not unique to this literature. It was also widely observed in the excluded studies. This creates an added challenge for those searching for relevant literature in that “sensory sensitivity” is nonspecific, referring to both multidimensional as well as unidimensional sensory domains and their relation to pain (including examples of QST). Thus, while “sensory sensitivity” was used by approximately half of all articles in this area, many did so without the use of an additional modifying term, much like many of the excluded studies. Our findings therefore suggest this is not particularly helpful for those searching for the MSS construct. However, when the modifying term, “sensory,” was used in conjunction with different core terms, ie, “sensory over-responsiveness” or “sensory hypervigilance,” the terms were more likely to be uniquely referring to a generalized sensitivity state and not only a unidimensional sensitivity.

While the core characteristic of this construct, ie, sensitivity to multiple sensory inputs, may seem obvious, it was sometimes unclear whether alternative core terms are used intentionally to reflect real, but possibly subtle, differences in meaning within the MSS construct umbrella. Conversely, some variation may simply reflect linguistic variations of no substantive consequence. Because few authors provided clear definitions of their MSS construct, it was difficult to discern the degree to which different authors' terminology could be considered synonyms. There were some instances of clear distinctions between terms, ie, sensory processing disorder is not a simply normal variation in sensory sensitivity but rather is a clinical designation falling at the far end of the possible MSS spectrum (eg, see Figure S3 in Supplemental Materials 2, http://links.lww.com/PR9/A252).^4–6,19,32,44^ Yet, despite it being a diagnostic category, it shares the essential core of sensitivity to multiple sensory stimuli and thus met the inclusion criteria for this scoping review. However, distinctions between construct definitions are less clear regarding the use of core terms such as “amplification,” “hypervigilance,” or “over-responsiveness” when compared with “sensitivity” or “hypersensitivity.” Our qualitative analysis picked up on subtle differences between terms such as these, but it is impossible to know whether these distinctions are intentional.

Even in the field of occupational therapy, where much of the early work in sensory processing has been performed, there has been some overlap between terminologies. A recent scoping review of sensory processing dysfunction in ASD found considerable heterogeneity in both the terminology used and the self-report instruments available.^26^ This suggests that even in the broader field of ASD, in which sensory processing dysfunction is a defining characteristic, clear definitions remain somewhat lacking. Schoen et al.^44^ raised concerns over inconsistent and varied use of terms in the autism arena more than a decade ago, indicating some authors differentiated sensory “defensiveness” as being generally limited to olfactory, auditory, and tactile stimuli but currently is largely used synonymously with over-responsivity. However, the phrase, “sensory defensiveness” was not noted as a primary terminology in this scoping review, which may indicate it has not been widely adopted by those interested in the relationship between MSS and pain. Nonetheless, it (and possibly other terms) may be present in the wider sensory literature.

The repeated omission of precise descriptions of MSS terms in this current body of literature may be a result of several possible explanations. First, it may be simply deemed unnecessary, as the terminology may seem at first to be self-explanatory. Second, the lack of clarity may be confounded by discipline-specific language norms that are assumed to be widely understood or accepted. Third, the consistent omission of construct descriptions might reflect the fundamental challenge of identifying a precise definition that extends beyond the use of similar descriptive terminology. However, the inclusion of at least a working operational definition of the construct being studied for each publication would help readers when interpreting these studies, especially given the often multidisciplinary interest in this growing area of pain research.

Interestingly, a description of the MSS term was often framed in terms of other MSS terms, either to clarify the construct *or* to identify related constructs or subcomponents. Thus, it was not uncommon to find the use of multiple terminologies within a single article.^20,25,50,55^ This may reflect the lack of clear definitions between terms, the attempt to generalize across terminologies, or a belief that these different terminologies have more in common than they are distinct.

Most studies focused on the heightened end of the possible MSS range, ie, *hyper*sensitivity to multiple sensory stimuli as opposed to *hypo*sensitivity, regardless of whether “hyper” was used as a modifying term. That is, the phrase “multisensory sensitivity” was frequently used interchangeably with multisensory *hyper*sensitivity. However, the full spectrum (hypo-sensitivity through hyper-sensitivity) was noted by a select few authors.^16,17,25,56^ As such, we developed a conceptual model of the full spectrum of MSS (see Figure S3, Supplemental Materials 2, http://links.lww.com/PR9/A252) to help add clarity when the use of language was not specific, showing the range from hypo-sensitivity to hyper-sensitivity. Indeed, from the broader occupational therapy discipline perspective when evaluating children and adults with sensory processing disorders, hyposensitivity is clearly differentiated (ie, using the AASP or its similar sensory profile assessments).^27,29^ However, this was less frequent in the remaining MSS and pain literature. While many of the included studies in this scoping review report on relations between hyper-MSS and pain, as suggested by one study, hypo-MSS also has relevance as a potential resilience indicator for multiple chronic overlapping pain conditions and might serve as a novel and useful research direction for future MSS-pain researchers.^56^ Of note, while conceptualizing an individual's generalized experience of MSS in terms of hypo-sensitivity and hyper-sensitivity may be useful, this is not synonymous with heightened or lessened sensitivity uniformly across all sensory domains. That is, while an individual may *on average* exhibit greater or lesser sensitivity to various daily stimuli, the specific affected domains may vary. This was recently noted in a case series of adolescent pain patients, where several with heightened MSS reported experiencing sensitivity in differing sensory domains.^54^

Throughout the included studies, heightened sensitivity was generally referring to whether the sensation(s) were reported in *negative terms* by the individual (eg, see Table 1 for numerous examples employing Likert scales) and less so on acuity (ie, ability to sense or intensity of a stimulus, eg, Body awareness Questionnaire^48^; Table 1). Similarly, “low” sensitivity was not typically used to represent low sensory acuity (poor eyesight, poor hearing, etc) but rather as not being bothered by or simply not noticing a sensation. Some instruments simply ask individuals if they experience sensitivity to stimuli without further definition of what “sensitivity to stimuli” means (eg, Adult sensory Questionnaire,^38^ Adult Autism Subthreshold Spectrum,^22^ Generalized Sensory Sensitivity/CMSI^58^ and Chronic Pain Questions^18^; Table 1), allowing each participant to evaluate their sensitivity in their own way. This brings to light the limitations of a self-report measure, as individuals self-reflect on their own sensitivities based on *what they presume* to be normal (and what they presume “sensitivity” to mean—higher intensity of a sensation vs experiencing sensations when others do not, etc). This brings us full circle to the question of underlying mechanism. Is a patient who reports sensitivity basing their trait of sensitivity predominately on a *belief* that they physically sense more or are more sensitive than what they presume others to feel (ie, psychological underpinning)? Or, are their sensations physically more intense, more long lasting, or occurring with less stimuli because of an “over-excited” CNS? Certainly, there is strong evidence linking the shared mechanisms in the processing of painful and nonpainful stimuli across several different neurophysiologic studies, as is nicely summarized in a recent review.^7^ Furthermore, a conceptual model linking the CNS processing of painful and nonpainful stimuli was proposed by these authors, highlighting imbalances between excitatory and inhibitory CNS responses as the underlying mechanism resulting in altered sensitivity to sensory input as well as pain as the manifestations.^7^ Self-report measures cannot distinguish between mechanisms or even estimate the degree to which each is a contributing factor. Both an underlying psychological or physiological (CNS based) underpinning can result in negative impacts and reduced quality of life for patients, yet different underlying factors may require different management strategies, even though as of yet targeted interventions remain ill-defined.^14,54^

The wide range of perspectives and disciplines converging on this topic of MSS and pain provides both advantages and challenges. The inherently diverse professional vocabularies and conceptualizations that underlie the authors' understanding and assessment of this construct can be viewed as a barrier to those entering this field from other disciplines. Yet, a benefit may be the extensive convergence of findings linking MSS (from whichever professional background it is described or assessed) and pain. For example, one of the first validated instruments found in the literature assessing the MSS construct in relation to pain is the SSAS. However, the SSAS was not designed with the a priori intention of measuring MSS per se as conceptualized today. Rather, the amplified sensory sensitivity as measured by the SSAS was construed as a psychological trait, related to hypochondriasis.^9^ This likely arose from Barsky psychiatric perspective, who published only shortly after the classic article first describing central modulation of pain was published, thus CNS modulation of sensory input was not widely recognized at that time.^59^ Whereas investigators from the occupational therapy discipline have been evaluating altered sensory processing for some time but again not with the specific intention of advancing our understanding of pain processing until the last decade.^4,6,27,29^ Other measures, such as the CMSI, Highly Sensitive Person Scale,^2,31^ etc., each represent in some way investigators from divergent origins converging to this common intersection, illustrating that assessments of nonpainful sensory sensitivity may be informative for, or relevant to, advancing our understanding of central pain processing.

As an evolving area in the field of pain science, there remains a lack of cross-disciplinary consensus regarding MSS assessment. For example, some instruments incorporate items related to behavioral consequences of heightened sensitivity, such as avoidance or coping strategies (eg, avoiding crowds), such as the sensory avoiding domain from the AASP.^28,29^ By contrast, other approaches focus more on perceptions of sensitivity, encompassing different sensory domains—such as used by the Multisensory Amplification Scale^55^ or the CMSI.^45,46,55^ This was highlighted by Wang et al.,^55^ the only study found which compared more than 2 MSS instruments in the same cohort. They reported moderate intercorrelations between several different assessments, further supporting a common construct, yet noted some clear distinctions between measures (ie, different sensory domains included, differential use of sensitivity vs avoidance items, etc). Other differences noted between instruments are whether they ask about how bothersome or annoying different sensory inputs may be (eg, SSAS, Multisensory Amplification Scale, Highly Sensitive Person Scale, AASP, etc.) with either graded or dichotomous options, whereas others ask the binary question of whether sensitivity to various inputs are present (ie, the CMSI subset or Generalized Sensory Sensitivity).^45,46^ Again, these assessments appear to produce relatively similar results (eg, correlations approximately 0.6^55^), suggesting the various assessment approaches are generally tapping into the same underlying construct. It is possible that subtle differences in assessment tools provide slightly different insights that may be of interest depending on the study goals, but to date, these assessments are not sufficiently well characterized to know one way or the other. We identified a proportion of studies that provided minimal information on their measurement approach, citing custom questions or interview (11%). This would generally not be considered acceptable rigor in other areas of pain science. Given the recent advances and availability of more tools in the past decade, we would expect a substantial decline in the use of custom, minimally defined measurement of MSS in future investigations.

One notable finding that emerged from this scoping review was that the MSS descriptions (and their underlying assessments) seem to somewhat mirror the biopsychosocial model of pain, in which there are psychological, biological/physiological, and social aspects to MSS. While the social aspects are the least clearly defined or assessed with MSS (as with pain), the overlap between largely psychological (ie, SSAS) and biological (ie, the physiology of CNS sensory processing) underpinnings is more readily discerned in the literature. While admittedly not precise or based on a well-defined rubric, given the lack of MSS definitions available, this conceptual model provides a starting framework to consider MSS in line with the analogous biopsychosocial model of pain. Arguably, the lack of social components of MSS may in fact be better represented in the “related-MSS” constructs described by others (eg, avoidant or seeking behaviors)^29,55^ or as the thematic impacts noted in this scoping review (Table 2). That is, while social factors are not a component of an individual's sensitivity, the resulting behavioral preferences may have notable social impacts on an individual. While several authors have proposed MSS may serve as a biomarker for central pain processing mechanisms, the social implications of MSS and its relationship to pain remains understudied yet may also be important. Thus, future research is needed to better characterize how MSS best fits within a biopsychosocial model.

While our primary purpose for this scoping review was to identify all MSS terms and assessments used in relation to pain, we recognize the limitation of this scoping process is the possibility that we missed relevant publications due to the wide use of similar terminology for unrelated investigations (eg, sensory sensitivity is used for both specific and generalized sensitivities). Furthermore, because sensory sensitivity has traditionally been a focus in the ASD field, there might be other relevant terminologies and surveys in that broader literature that has not yet infiltrated into the pain domain that may be relevant. Thus, our scoping review would not be able to capture these based on the inclusion and exclusion criteria.

In summary, the consistent findings and/or conclusions from across studies linking aspects of pain with some assessment of MSS suggests that these cross-discipline terminologies show some degree of consensus, despite the highly varying use of core and modifying terms. Furthermore, the convergence from multiple disciplines into this area of study, while a likely contributor to the observed variability in the use of MSS terminology, exemplifies the potential impact and significance of this emerging area of pain science. Indeed, based on the journals in which included studies were published, MSS in the context of pain is relevant to medicine and psychology disciplines in addition to the more specialized areas of pain science. The insights gained from this review ultimately may facilitate the exchange of relevant information across disciplines by promoting awareness of the terminology used to date and identifying gaps in definitions for the various terms used. Based on our findings, we would suggest the following recommendations for improved precision in future MSS and pain publications: (1) clearly define the “MSS” term(s) used and clearly indicate the multinature or generalized nature of the sensitivity within the term itself, (2) specify where along the spectrum (hypo-sensitivity vs hyper-sensitivity) the study is focused, and (3) specify the sensory domains included in the MSS assessment.

## Disclosures

The authors have no conflicts of interest to declare.

This study was funded in part by an Iowa Center for Research by Undergraduates (ICRU) Fellowship (HD) from the Office of Undergraduate Research at the University of Iowa. The results of this study do not reflect the perspectives or imply endorsement by the ICRU.

## Appendix A. Supplemental digital content

Supplemental digital content associated with this article can be found online at http://links.lww.com/PR9/A252.

## Supplementary Material
