# Supplementary material for: Multisensory sensitivity in relation to pain: a scoping review of terminology and assessment
Source: Pain Rep. 2024 Oct 25;9(6):e1193. doi: 10.1097/PR9.0000000000001193 (PMC11519410; doi:10.1097/PR9.0000000000001193)
Supplement: Supplementary file 1 [file painreports-9-e1193-s001.pdf]

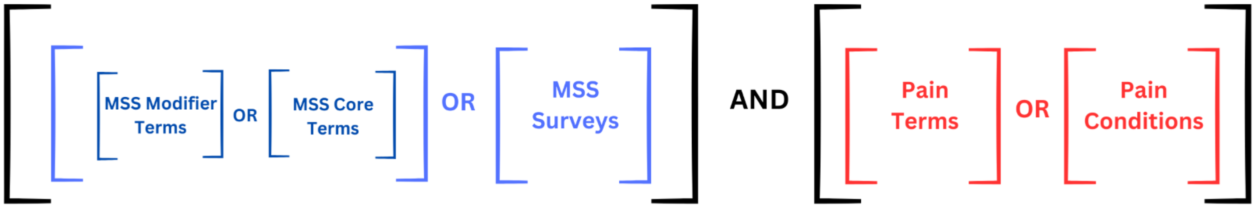

Figure S1. Conceptual visualization of the search strategy used to identify all possible MSS-pain papers. The left section in blue targeted MSS papers and the right section in red targeted pain-related papers. The two search strings were then combined with the "AND" Boolean operator to find those studies satisfying both comprehensive search strings.

Table S1. List of abbreviations used.

Abbreviation	Definition
AASP	Adolescent & Adult Sensory Profile
AdAS	Adult Autism Subthreshold Spectrum
ASD	Autism Spectrum Disorder
ASQ	Adult Sensory Questionnaire
BAQ	Body Awareness Questionnaire
BVS	Body Vigilance Scale
CCP	Cardiac Chest Pain
CFS	Chronic Fatigue Syndrome
CLBP	Chronic Low Back Pain
CMSI	Complex Medical Symptoms Inventory
CNS	Central Nervous System
COPC	Chronic Overlapping Pain Conditions
CPQ	Chronic Pain Questions
CRPS	Complex Regional Pain Syndrome
F	Female
FM	Fibromyalgia
HC	Healthy Controls
HSCS	Highly Sensitive Child Scale
HSPS	Highly Sensitive Persons Scale
IBD	Inflammatory Bowel Disease
IBS	Irritable Bowel Syndrome
IC	Interstitial Cystitis
LBP	Low Back Pain
M	Male
MDD	Major Depressive Disorder
MFP	Myofascial Face Pain
MSAS	Multisensory Amplification Scale

Table S1 (cont'd). List of abbreviations used.

Abbreviation	Definition
MSHS	Multisensory Hypersensitivity
MSS	Multisensory Sensitivity
NCCP	Non-cardiac Chest Pain
NSCLBP	Non-specific Chronic Low Back Pain
PANS	Pediatric Acute-onset Neuropsychiatric Syndrome
PPPD	Persistent Postural Perceptual Dizziness
PPTH	Persistent Post-Traumatic Headache
PRISMA-Scr	Preferred Reporting Items for Systematic Reviews and Meta-analysis Extension for Scoping Reviews
Psych	Psychiatric Patients
RA	Rheumatoid Arthritis
SH	Sensory Hypersensitivity
SHS	Sensory Hypersensitivity Scale
SMD	Sensory Modulation Disorder
SOR	Sensory Over-responsiveness/Over-responsivity
SP	Sensory Profile Questionnaire
SPQ	Sensory Perception Quotient
SPSI	Sensory Processing Scale Inventory
SRQ	Sensory Responsiveness Questionnaire
SRQ-IS	Sensory Responsiveness Questionnaire - Intensity Scale
SSAS	Somatosensory Amplification Scale
SSP	Short Sensory Profile
SSP2	Short Sensory Profile 2
TMD	Temporomandibular Disorder

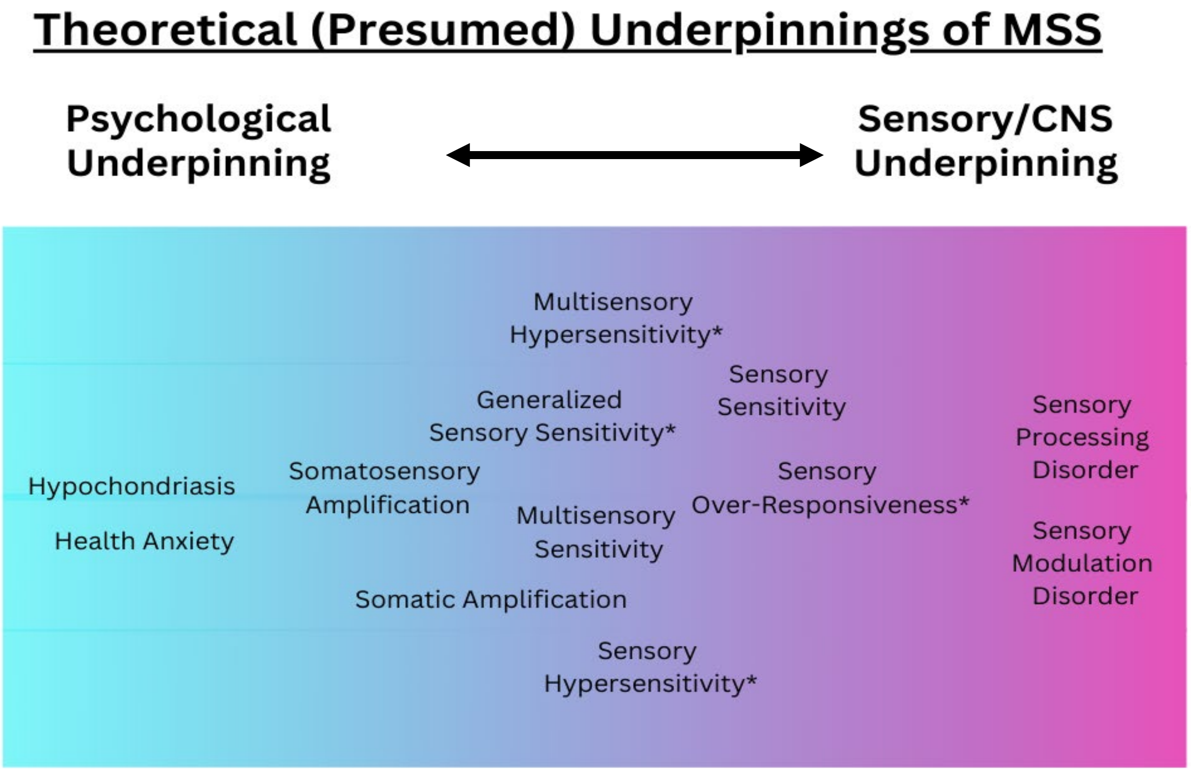

Figure S2. Theoretical (presumed) underpinnings of MSS as identified from descriptions in, and thematic analysis of, the 92 included publications. This spectrum is a conceptual model meant to illustrate the underlying biopsychosocial aspect of MSS, like pain, although notably the social component remains lacking to date. Two different underpinnings of MSS emerged in the literature: a predominately “psychological“ perspective and a “biological” perspective that focused on sensory or central nervous system (CNS) factors. Note, a mechanistic model linking neurophysiologic CNS factors with sensory sensitivity is proposed by Bar-Shalita et al. (Frontiers in Integrated Neuroscience, 2019). As authors did not frequently or explicitly discuss MSS underpinnings in their papers, we approximated the relative placement for each MSS term within this spectrum based on underlying descriptions of MSS, but note this is not meant to reflect a linear scale. The use of a spectrum illustrates that these two different underpinnings are not discrete categories, but rather represent a range of different authors’ characterization of MSS. *Indicates that at least 1 author’s description of MSS included pain as a component in some manner.

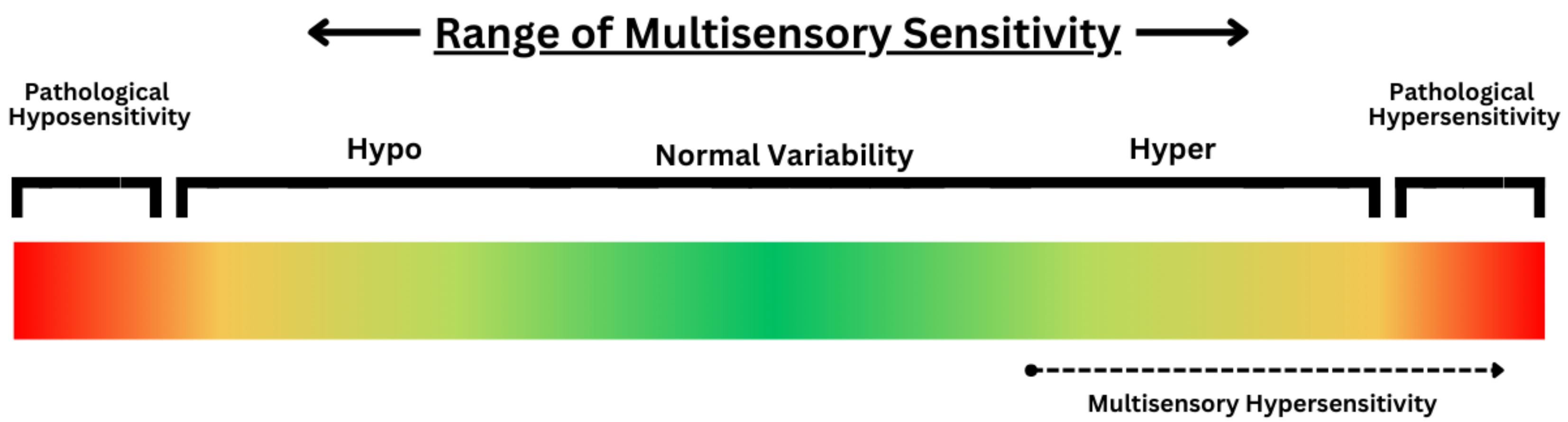

Figure S3. This conceptual model is an inexact, general representation of the full multisensory sensitivity (MSS) spectrum, from less ("hyposensitive") to more ("hypersensitive") to multiple sensory inputs. Note, however, that this general sensitivity is not synonymous with heightened or lessened sensitivity uniformly across all sensory domains. That is, while an individual may *on average* exhibit greater or lesser sensitivity to various daily stimuli, this does not signify that all sensory domains will be equally affected. For example, one may be particularly sensitive to a select few inputs and less so to others. Thus, the assessment of multisensory sensitivity cannot be identified simply from a single sensory domain and is best reflected when considering multiple different domains. At the extremes of the continuum, the variation in sensitivity may be considered pathological, i.e., a clinical disorder. However, there is a wide range of normal variation between those extremes. Much of the recent pain literature is exploring these intermediate ranges in MSS, with a heavy emphasis on the hypersensitivity portion of the spectrum.

Table S2. All included MSS and pain studies (n = 92), ordered alphabetically by first author.

First Author (date)	Journal	Journal Type	Population	Age Group	Sex	MSS Term*	MSS Surveys Used	Ref
Ak (2004)	Pain Clinic	Pain	Chronic pain, healthy controls (HC)	Adult	Both	Somatosensory amplification	SSAS	[1]
Akay (2022)	Eur Res J	General research	Fibromyalgia (FM)	Adult	Female	Somatosensory amplification	SSAS	[2]
Amery (1988)	Ital J Neurol Sci	Neurosci/ Brain Sci	Migraine	Adult	Both	Sensory sensitivity/ sensory changes	Custom	[3]
Assayag (2020)	Pain Medicine	Pain	Substance Use Disorder, HC	Adult	Both	Sensory responsiveness	SRQ-IS	[4]
Barke (2014)	J Pain Res	Pain	HC	Child	Both	Somatosensory amplification	SSAS	[11]
Bar-Shalita (2009)	Physiol Behav	Behavior/ Cognitive/ Psych	Sensory Modulation Disorder (SMD), HC	Child	Both	Sensory over-responsiveness & Sensory Modulation Disorder (SMD)	SSP, SP	[5]
Bar-Shalita (2012)	Disabil Rehabil	Rehabilitation	SMD, HC	Adult	Both	Somatosensory sensitivity	SRQ-IS	[6]
Bar-Shalita (2014)	Exp Brain Res	Neurosci/Brain Sci	SMD, HC	Adult	Both	Sensory over-responsivity & SMD	SRQ-IS	[7]
Bar-Shalita (2018)	PLOS One	General research	Complex Regional Pain Syndrome (CRPS), HC	Adult	Both	Sensory over-responsivity & SMD	SRQ-IS	[8]
Bar-Shalita (2020)	Brain Sci	Neurosci/ Brain Sci	Sensory over-responsive (SOR), HC	Adult	Female	Sensory over-responsivity	SRQ-IS	[10]
Bar-Shalita (2020)	Frontiers in Integrative Neuroscience	Neurosci/ Brain Sci	Community-dwelling	Adult	Both	Sensory over-responsiveness	SRQ-IS	[9]
Batmaz (2014)	Myopain	Pain	CRPS, arm/hand tendon injury, HC	Adult	Both	Somatosensory amplification	SSAS	[12]
Carmassi (2021)	Clin Exp Rheumatol	Medical	FM	Adult	Both	Multisensory hypersensitivity	AdAS subsection	[13]

Supplementary Materials - MSS and Pain Scoping Review

First Author (date)	Journal	Journal Type	Population	Age Group	Sex	MSS Term*	MSS Surveys Used	Ref
Chow (2019)	Clin Oral Investig	Dental	Temporomandibular disorder (TMD), HC	Adult	Both	Somatosensory amplification	SSAS	[14]
Ciaramella (2021)	Scand J Pain	Pain	FM, Chronic pain	Adult	Both	Somatosensory amplification *also somatic amplification	SSAS	[15]
Ciaramella (2022)	Scand J Pain	Pain	Chronic pain, HC	Adult	Both	Interoceptive sensitivity/ somatic amplification	SSAS	[16]
Ciaramella (2023)	Am J Clin Hypn	Behavior/ Cognitive/ Psych	Chronic pain, HC	Adult	Both	Somatosensory amplification	SSAS	[17]
Clark (2018)	J Bodyw Mov Ther	Rehabilitation	Chronic low back pain (CLBP)	Adult	Both	Somatosensory hypersensitivity	AASP	[18]
Clark (2019)	Braz J Phys Ther	Rehabilitation	CLBP	Adult	Both	Sensory sensitivity/hypersensitivity	AASP, Custom	[19]
Clark (2019)	Pain Physician	Pain	CLBP	Adult	Both	Sensory hypersensitivity	AASP	[20]
Clark (2019)	Pain Pract	Pain	CLBP	Adult	Both	Sensory sensitive, trait sensory hypersensitivity	CSI, AASP	[21]
Costa (2016)	J Oral Facial Pain Headache	Headache	HC	Adult	Both	Somatosensory amplification	SSAS	[22]
Coyne (2017)	Postgrad Med	Medical	Neuropathic pain, FM, TMD, IBS, IC, CFS	Adult	Both	Sensory hypersensitivity	CPQ subsection	[23]
Crofton (2022)	Burns	Medical	Burn	Adult	Both	Sensory sensitivity	AASP	[24]
Den Boer (2021)	Pain Pract	Pain	Health care practitioners	Adult	Both	Sensory hypersensitivity	SHS	[25]
Dixon (2016)	J Behav Med	Behavior/ Cognitive/ Psych	LBP, HC	Adult	Both	Sensory hypersensitivity	SHS, HSPS	[26]
Dorris (2022)	Front Pain Res (Lausanne)	Pain	LBP, HC	Adult	Both	sensory hypersensitivity	SPQ	[27]

First Author (date)	Journal	Journal Type	Population	Age Group	Sex	MSS Term*	MSS Surveys Used	Ref
Duale (2021)	Pain Physician	Pain	HC	Adult	Both	Sensory over-responsivity	Custom	[28]
Dumkrieger (2022)	Front Pain Res (Lausanne)	Pain	Migraine, persistent post-traumatic headache (PPTH)	Adult	Both	Sensory hypersensitivity	Multi**	[29]
Engel-Yeger (2011)	Am J Occ Ther	Rehabilitation	HC	Adult	Both	Sensory over-responsivity	AASP	[30]
Frankovich (2015)	J Child Adolesc Psychopharmacol	Behavior/ Cognitive/ Psych	Neuropsych syndrome (PANS)	Child	Both	Sensory amplification	Custom	[31]
Genizi (2019)	Front Neurol	Neurosci/ Brain Sci	Migraine, HC	Child	Both	Sensory processing disorder	SSP	[32]
Genizi (2020)	J Headache Pain	Headache	Migraine, HC	Adolescent	Both	Sensory sensitivity ("enhanced sensory sensitivity")	AASP	[33]
Ginzburg (2018)	J Pers Disord	Headache	Borderline personality disorder, HC	Adult	Female	Body awareness, interoceptive awareness, interoceptive sensitivity,	BAQ	[34]
Granovsky (2018)	J Pain	Pain	Migraine	Adult	Female	Sensory responsiveness, Sensory over-responsiveness	SRQ-IS	[35]
Grant (2022)	Mol Autism	Autism	Autism Spectrum Disorder (ASD)	Adult	Both	Sensory sensitivity	SPQ	[36]
Graper (2022)	Physiother Theory Pract	Rehabilitation	CLBP	Adult	Both	Sensory sensitivity	AASP	[37]
Gregory (2000)	Psychosomatics	Behavior/ Cognitive/ Psych	Psychiatric patients (psych), chronic pain	Adult	Both	Somatosensory amplification	SSAS	[38]
Gregory (2005)	Ann Clin Psychiatry	Behavior/ Cognitive/ Psych	Psych	Adult	Both	Somatosensory amplification	SSAS	[39]
Guclu (2012)	Turk Neurosurg	Neurosci/ Brain Sci	CLBP	Adult	Both	Somatosensory amplification	SSAS	[40]

First Author (date)	Journal	Journal Type	Population	Age Group	Sex	MSS Term*	MSS Surveys Used	Ref
Hacimusalar (2020)	Ind J Surg	Medical	Mastalgia	Adult	Female	Somatosensory amplification	SSAS	[41]
Haider (2023)	Pain	Pain	FM, COVID-19, CFS	Adult	Both	Multisensory sensitivity	MSAS-7	[42]
Hanna (2020)	Cephalalgia	Headache	PPTH, migraine, HC	Adult	Both	Sensory hypersensitivity	Multi**	[43]
Hoffman (2023)	Pain	Pain	ASD, HC	Adult	Both	Sensory hypersensitivity	SRQ-IS	[44]
Inal (2020)	Somatosens Mot Res	Other	FM	Adult	Both	Sensory processing	AASP	[45]
Kaufman (2022)	Front Neurosci	Neurosci/ Brain Sci	Misophonia, HC	Adult	Both	Sensory over-responsiveness	SRQ-IS	[46]
Kim (2020)	J Headache Pain	Headache	PPTH, migraine, HC	Adult	Both	Sensory hypersensitivity	Multi**	[47]
Koechlin (2023)	Pain Rep	Pain	Chronic pain	Adoles cent	Both	Sensory processing sensitivity	HSC	[48]
Kosturek (1998)	Psychosomatics	Behavior/ Cognitive/ Psych	Chronic pain, psych	Adult	Both	Somatic amplification	SSAS	[49]
Koteles (2016)	J Health Psychol	Behavior/ Cognitive/ Psych	General practitioner patients, HC	Adult	Both	Somatosensory amplification	SSAS	[50]
Kroner-Herwig (2017)	Adolesc Health Med Ther	Medical	Headache, LBP, HC	Adult	Both	Somatosensory amplification	Custom	[51]
Labus (2023)	Pain	Pain	Irritable Bowel syndrome (IBS), HC	Adult	Both	Multisystem sensory sensitivity	CMSI subsection	[52]
Leveque (2020)	Rev Neurol (Paris)	Neurosci/ Brain Sci	Migraine, HC	Adult	Both	Sensory hypersensitivity	Multi**	[53]
Lo (2022)	J Headache Pain	Headache	Migraine	Adult	Both	Sensory sensitivity	Custom	[54]

First Author (date)	Journal	Journal Type	Population	Age Group	Sex	MSS Term*	MSS Surveys Used	Ref
López-Solá (2014)	Arthritis Rheumatol	Medical	FM, HC	Adult	Female	Multisensory hypersensitivity	AASP	[55]
Martins (2006)	J Pain	Pain	Migraine, tension-type headache,	Adult	Both	Sensory intolerance	Custom	[56]
Meredith (2015)	Am J Occ Ther	Rehabilitation	HC	Adult	Both	Sensory sensitivity	AASP	[57]
Midenfjord (2021)	Neurogastroent erol Motil	Medical	IBS, IBD, HC	Adult	Both	hypersensitivity (for other stimuli)	HSPS	[58]
Naliboff (2022)	J Urol	Medical	Pelvic pain patients	Adult	Both	Sensory sensitivity	CMSI subsection	[59]
Ogrodniczuk (2018)	Psychiatry Res	Behavior/ Cognitive/ Psych	Psych	Adult	Both	Somatosensory amplification	SSAS	[60]
Ozsoy (2021)	Erciyes Medical Journal	Medical	Chronic pain, HC	Adult	Both	Somatosensory amplification	SSAS	[61]
Park (2015)	J Headache Pain	Headache	Migraine	Adult	Both	Sensory hypersensitivity	Multi**	[62]
Pearl (2020)	Headache	Headache	Migraine	Adult	Both	Sensory, Multisensory, or multi-modal hypersensitivity	Multi**	[63]
Perkins (2016)	Atten Percept Psychophys	Behavior/ Cognitive/ Psych	HC	Adult	Both	Cross-modal sensitivity	AASP	[64]
Peters (2000)	Pain	Pain	FM, HC	Adult	Female	Hypervigilance	BVS	[65]
Pierce (2021)	Ann Behav Med	Behavior/ Cognitive/ Psych	Knee osteoarthritis	Adult	Both	Generalized sensory sensitivity	CMSI items /GSS	[66]
Powell (2020)	J Neurol	Neurosci/ Brain Sci	Vestibular, HC	Adult	Both	Multisensory sensitivity	AASP	[67]
Price (2021)	Headache	Headache	HC	Adult	Both	Sensory hypersensitivity	AASP	[68]

First Author (date)	Journal	Journal Type	Population	Age Group	Sex	MSS Term*	MSS Surveys Used	Ref
Rao (2023)	Reg Anesth Pain Med	Medical	CLBP	Adult	Both	Sensory hypersensitivity	SHS	[69]
Raphael (2000)	Pain Med	Pain	Face pain/TMD, HC	Adult	Female	Somatosensory amplification	SSAS modified	[70]
Rost (2017)	J Psychosom Res	Behavior/ Cognitive/ Psych	FM, HC	Adult	Both	Generalized hypervigilance	BVS	[71]
Ryu (2021)	J Pers Med	Medical	SOR, HC	Adult	Both	Sensory over-responsivity	SRQ-IS	[72]
Sayar (2005)	Psychosomatics	Behavior/ Cognitive/ Psych	FM, major depressive disorder (MDD)	Adult	Both	Somatosensory amplification	SSAS	[73]
Schrepf (2018)	Pain	Pain	Chronic pelvic pain, General chronic pain, HC	Adult	Both	Generalized sensory sensitivity	CMSI items	[74]
Schrepf (2023)	Pain	Pain	Dysmenorrhea	Adult	Female	Generalized sensory sensitivity	CMSI items/GSS	[75]
Schroeder (2012)	Psychosom Med	Medical	Non-cardiac and cardiac chest pain	Adult	Both	Somatic amplification	SSAS	[76]
Schroeder (2014)	J Behav Ther Exp Psychiatry	Behavior/ Cognitive/ Psych	Chest pain (NCCP & CCP), HC	Adult	Both	Somatosensory amplification	SSAS	[77]
Seo (2019)	Neurol Sci	Neurosci/ Brain Sci	SMD, HC	Adult	Both	Sensory hypersensitivity	Custom	[78]
Sinclair (2019)	Clin J Pain	Pain	Chronic pain	Adult	Both	Sensory over-responsiveness	AASP	[79]
Sinclair (2020)	Am J Occup Ther	Rehabilitation	Chronic pain	Adult	Both	Sensory over-responsiveness, sensory modulation disorder (SMD)	AASP	[80]
Stewart (1994)	Obstet Gynecol	Medical	Vulvodynia, HC	Adult	Female	Somatosensory amplification	SSAS	[81]

First Author (date)	Journal	Journal Type	Population	Age Group	Sex	MSS Term*	MSS Surveys Used	Ref
Subasi Tekintas (2022)	Noro Psikiyatr Ars	Other	MDD, FM, HC	Adult	Both	Somatosensory amplification	SSAS	[82]
Sullivan (2014)	Autism	Autism	ASD	Adult	Both	Sensory hyperreactivity, general neuronal hyperexcitability	SPSI - SOR subsection	[83]
Suzuki (2021)	J Headache Pain	Headache	Migraine	Adult	Both	Sensory hypersensitivity	Custom	[84]
Ten Brink (2020)	Pain	Pain	CRPS, FM, Chronic pain, HC	Adult	Both	Sensory sensitivity	Custom	[85]
Vrbanovic (2022)	Acta Odontol Scand	Dental	TMD, HC	Adult	Both	Somatosensory amplification	SSAS	[86]
Wang (2020)	J Pain Res	Pain	HC	Adult	Both	Multisensory sensitivity	SSAS -subset	[87]
Wang (2022)	J Pain	Pain	Community-dwelling	Adult	Both	Multisensory sensitivity	MSAS	[88]
Wang (2023)	Pain	Pain	FM, Migraine, LBP, HC	Adult	Both	Multisensory sensitivity	MSAS	[89]
Weissman-Fogel (2018)	Pain Pract	Pain	SMD, HC	Adult	Both	Multisensory hypersensitivity	SQR-IS	[90]
Willbarger (2011)	Arch Phys Med Rehabil	Medical	FM, RA, HC	Adult	Female	Multisensory hypersensitivity	Multi**	[91]
Yavuz (2013)	J Headache Pain	Headache	Migraine, HC	Adult	Both	Somatic amplification, somatosensory amplification	SSAS	[92]

Notes: Multi** = MSS assessed using a combination of multiple single sensory sensitivity surveys such as phonophobia, photophobia, etc.

Included Manuscripts:

[1] Ak I, Sayar K, Yontem T. Alexithymia, somatosensory amplification and counter-dependency in patients with chronic pain. Pain Clinic 2004;16(1):43-51.
[2] Akay E, Şenormancı Ö, Eröksüz R, Şenormancı G, Demirci OO. The effect of psychopathology on quality of life and disability in patients with fibromyalgia. European Research Journal 2022;8(1):103-110.
[3] Amery WK, Waelkens J, Vandenbergh V. The sensorium of the migraineur. Italian journal of neurological sciences 1988;9(6):539-545.
[4] Assayag N, Bonneh Y, Parush S, Mell H, Kaplan Neeman R, Bar-Shalita T. Perceived Sensitivity to Pain and Responsiveness to Non-noxious Sensation in Substance Use Disorder. Pain Med 2020;21(9):1902-1912.
[5] Bar-Shalita T, Vatine JJ, Seltzer Z, Parush S. Psychophysical correlates in children with sensory modulation disorder (SMD). Physiol Behav 2009;98(5):631-639.
[6] Bar-Shalita T, Vatine JJ, Parush S, Deutsch L, Seltzer Z. Psychophysical correlates in adults with sensory modulation disorder. Disabil Rehabil 2012;34(11):943-950.
[7] Bar-Shalita T, Vatine JJ, Yarnitsky D, Parush S, Weissman-Fogel I. Atypical central pain processing in sensory modulation disorder: Absence of temporal summation and higher after-sensation. Experimental Brain Research 2014;232(2):587-595.
[8] Bar-Shalita T, Livshitz A, Levin-Meltz Y, Rand D, Deutsch L, Vatine JJ. Sensory modulation dysfunction is associated with Complex Regional Pain Syndrome. PLoS One 2018;13(8):e0201354.
[9] Bar-Shalita T, Cermak SA. Multi-sensory Responsiveness and Personality Traits Predict Daily Pain Sensitivity. Front Integr Neurosci 2019;13:77.
[10] Bar-Shalita T, Ben-Ziv N, Granovsky Y, Weissman-Fogel I. An exploratory study testing autonomic reactivity to pain in women with sensory over-responsiveness. Brain Sciences 2020;10(11):1-9.
[11] Barke A, Gaßmann J, Kröner-Herwig B. Cognitive processing styles of children and adolescents with headache and back pain: A longitudinal epidemiological study. Journal of Pain Research 2014;7:405-414.
[12] Batmaz I, Dilek B, Sariyildiz MA, Erbatur S, Bez Y, Yazmalar L, Bozkurt M, Çevik R. Depressive and anxious temperaments in patients with complex regional pain syndrome secondary to tendon injury. Journal of Musculoskeletal Pain 2014;22(2):139-144.
[13] Carmassi C, Cordone A, Ciapparelli A, Bertelloni CA, Barberi FM, Foghi C, Pedrinelli V, Dell'Oste V, Bazzichi L, Dell'Osso L. Adult Autism Subthreshold spectrum correlates to Post-traumatic Stress Disorder spectrum in patients with fibromyalgia. Clinical and Experimental Rheumatology 2021;39(3).
[14] Chow JC, Cioffi I. Effects of trait anxiety, somatosensory amplification, and facial pain on self-reported oral behaviors. Clin Oral Investig 2019;23(4):1653-1661.
[15] Ciaramella A, Silvestri S, Pozzolini V, Federici M, Carli G. A retrospective observational study comparing somatosensory amplification in fibromyalgia, chronic pain, psychiatric disorders and healthy subjects. Scand J Pain 2021;21(2):317-329.
[16] Ciaramella A, Pozzolini V, Scatena E, Carli G. Can interoceptive sensitivity provide information on the difference in the perceptual mechanisms of recurrent and chronic pain? Part I. A retrospective clinical study related to multidimensional pain assessment. Scandinavian Journal of Pain 2022.
[17] Ciaramella A. Hypnotic analgesia in chronic pain: role of psychopathology and alexithymia. The American journal of clinical hypnosis 2023:1-15.

[18] Clark JR, Yeowell G, Goodwin PC. Trait anxiety and sensory processing profile characteristics in patients with non-specific chronic low back pain and central sensitisation - A pilot observational study. J Bodyw Mov Ther 2018;22(4):909-916.

[19] Clark JR, Goodwin PC, Yeowell G. Exploring the pre-morbid contexts in which central sensitisation developed in individuals with non-specific chronic low back pain. A qualitative study. Braz J Phys Ther 2019;23(6):516-526.

[20] Clark JR, Nijs J, Smart K, Holmes P, Yeowell G, Goodwin PC. Prevalence of Extreme Trait Sensory Profiles and Personality Types in Nonspecific Chronic Low Back Pain with Predominant Central Sensitization: Secondary Analysis of an International Observational Study. Pain Physician 2019;22(3):E181-e190.

[21] Clark JR, Nijs J, Yeowell G, Holmes P, Goodwin PC. Trait Sensitivity, Anxiety, and Personality Are Predictive of Central Sensitization Symptoms in Patients with Chronic Low Back Pain. Pain Pract 2019;19(8):800-810.

[22] Costa YM, Baad-Hansen L, Bonjardim LR, Rodrigues Conti PC, Svensson P. Is the Nociceptive Blink Reflex Associated with Psychological Factors in Healthy Participants? Journal of oral & facial pain and headache 2016;30(2):120-126.

[23] Coyne KS, Currie BM, Donevan S, Cappelleri JC, Hegeman-Dingle R, Abraham L, Thompson C, Sadosky A, Brodsky M. Discriminating between neuropathic pain and sensory hypersensitivity using the Chronic Pain Questions (CPQ). Postgrad Med 2017;129(1):22-31.

[24] Crofton E, Meredith P, Gray P, Strong J. Sensory processing and detection thresholds of burn-injured patients: A comparison to normative data. Burns (03054179) 2022;48(7):1590-1598.

[25] den Boer C, Terluin B, van der Wouden JC, Blankenstein AH, van der Horst HE. Tests for central sensitization in general practice: a Delphi study. BMC Fam Pract 2021;22(1):206.

[26] Dixon EA, Benham G, Sturgeon JA, Mackey S, Johnson KA, Younger J. Development of the Sensory Hypersensitivity Scale (SHS): a self-report tool for assessing sensitivity to sensory stimuli. Journal of behavioral medicine 2016;39(3):537-550.

[27] Dorris ER, Maccarthy J, Simpson K, McCarthy GM. Sensory Perception Quotient Reveals Visual, Scent and Touch Sensory Hypersensitivity in People With Fibromyalgia Syndrome. Front Pain Res (Lausanne) 2022;3:926331.

[28] Dualé C, Leray V, Giron F, Boulliau S, Macian N, Ruscheweyh R, Dubray C, Giraudet F. The added value of sensitivity to nonnoxious stimuli to predict an individual’s sensitivity to pain. Pain Physician 2021;24(6):E783-E794.

[29] Dumkrieger G, Chong CD, Ross K, Berisha V, Schwedt TJ. The value of brain MRI functional connectivity data in a machine learning classifier for distinguishing migraine from persistent post-traumatic headache. Front Pain Res (Lausanne) 2022;3:1012831.

[30] Engel-Yeger B, Dunn W. Relationship between pain catastrophizing level and sensory processing patterns in typical adults. The American Journal of Occupational Therapy 2011;65(1):e1-e10.

[31] Frankovich J, Thienemann M, Pearlstein J, Crable A, Brown K, Chang K. Multidisciplinary clinic dedicated to treating youth with pediatric acute-onset neuropsychiatric syndrome: presenting characteristics of the first 47 consecutive patients. J Child Adolesc Psychopharmacol 2015;25(1):38-47.

[32] Genizi J, Halevy A, Schertz M, Osman K, Assaf N, Segal I, Srugo I, Kessel A, Engel-Yeger B. Sensory processing difficulties correlate with disease severity and quality of life among children with migraine. Frontiers in Neurology 2019;10.

[33] Genizi J, Halevy A, Schertz M, Osman K, Assaf N, Segal I, Srugo I, Kessel A, Engel-Yeger B. Sensory processing patterns affect headache severity among adolescents with migraine. Journal of Headache & Pain 2020;21(1):1-7.

[34] Ginzburg K, Biran I, Aryeh IG, Tsur N, Defrin R. Pain Perception and Body Awareness Among Individuals With Borderline Personality Disorder. Journal of personality disorders 2018;32(5):618-635.
[35] Granovsky Y, Shor M, Shifrin A, Sprecher E, Yarnitsky D, Bar-Shalita T. Assessment of Responsiveness to Everyday Non-Noxious Stimuli in Pain-Free Migraineurs With Versus Without Aura. J Pain 2018;19(8):943-951.
[36] Grant S, Norton S, Weiland RF, Scheeren AM, Begeer S, Hoekstra RA. Autism and chronic ill health: an observational study of symptoms and diagnoses of central sensitivity syndromes in autistic adults. Molecular Autism 2022;13(1).
[37] Gräper PJ, Clark JR, Thompson BL, Hallegraeff JM. Evaluating sensory profiles in nociplastic chronic low back pain: a cross-sectional validation study. Physiotherapy theory and practice 2022;38(10):1508-1518.
[38] Gregory RJ, Manring J, Berry SL. Pain location and psychological characteristics of patients with chronic pain. Psychosomatics 2000;41(3):216-220.
[39] Gregory RJ, Manring J, Wade MJ. Personality traits related to chronic pain location. Ann Clin Psychiatry 2005;17(2):59-64.
[40] Guclu DG, Guclu O, Ozaner A, Senormanci O, Konkan R. The relationship between disability, quality of life and fear-avoidance beliefs in patients with chronic low back pain. Turk Neurosurg 2012;22(6):724-731.
[41] Hacimusalar Y, Talih T, Karaaslan O. How Do Health Anxiety, Somatosensory Amplification, and Depression Levels Relate to Non-cyclical Mastalgia? A Case–Control Study. Indian Journal of Surgery 2020;82(4):578-584.
[42] Haider S, Janowski AJ, Lesnak JB, Hayashi K, Dailey DL, Chimenti R, Frey-Law LA, Sluka KA, Berardi G. A comparison of pain, fatigue, and function between post-COVID-19 condition, fibromyalgia, and chronic fatigue syndrome: a survey study. Pain 2023;164(2):385-401.
[43] Hanna JJ, Chong CD, Dumkrieger GM, Ross KB, Schwedt TJ. Sensory hypersensitivities in those with persistent post-traumatic headache versus migraine. Cephalalgia Reports 2020;3.
[44] Hoffman T, Bar-Shalita T, Granovsky Y, Gal E, Kalingel-Levi M, Dori Y, Buxbaum C, Yarovinsky N, Weissman-Fogel I. Indifference or hypersensitivity? Solving the riddle of the pain profile in individuals with autism. Pain 2023;164(4):791-803.
[45] İnal Ö, Aras B, Salar S. Investigation of the relationship between kinesiophobia and sensory processing in fibromyalgia patients. Somatosens Mot Res 2020;37(2):92-96.
[46] Kaufman AE, Weissman-Fogel I, Rosenthal MZ, Neeman RK, Bar-Shalita T. Opening a window into the riddle of misophonia, sensory over-responsiveness, and pain. Front Neurosci 2022;16:907585.
[47] Kim S-K, Chong CD, Dumkrieger G, Ross K, Berisha V, Schwedt TJ. Clinical correlates of insomnia in patients with persistent post-traumatic headache compared with migraine. Journal of Headache & Pain 2020;21(1):1-10.
[48] Koechlin H, Donado C, Locher C, Kossowsky J, Lionetti F, Pluess M. Sensory processing sensitivity in adolescents reporting chronic pain: an exploratory study. Pain Rep 2023;8(1):e1053.
[49] Kosturek A, Gregory RJ, Sousou AJ, Trief P. Alexithymia and somatic amplification in chronic pain. Psychosomatics 1998;39(5):399-404.
[50] Köteles F, Doering BK. The many faces of somatosensory amplification: The relative contribution of body awareness, symptom labeling, and anxiety. Journal of Health Psychology 2016;21(12):2903-2911.
[51] Kröner-Herwig B, Gorbunova A, Maas J. Predicting the occurrence of headache and back pain in young adults by biopsychological characteristics assessed at childhood or adolescence. Adolesc Health Med Ther 2017;8:31-39.

[52] Labus JS, Wang C, Mayer EA, Gupta A, Oughourlian T, Kilpatrick L, Tillisch K, Chang L, Naliboff B, Ellingson BM. Sex-specific brain microstructural reorganization in irritable bowel syndrome. Pain 2023;164(2):292-304.
[53] Lévêque Y, Masson R, Fornoni L, Moulin A, Bidet-Caulet A, Caclin A, Demarquay G. Self-perceived attention difficulties are associated with sensory hypersensitivity in migraine. Rev Neurol (Paris) 2020;176(10):829-838.
[54] Lo SH, Gallop K, Smith T, Powell L, Johnston K, Hubig LT, Williams E, Coric V, Harris L, L'Italien G, Lloyd AJ. Real-World experience of interictal burden and treatment in migraine: a qualitative interview study. Journal of Headache & Pain 2022;23(1):1-10.
[55] Lõpez-Solà M, Pujol J, Wager TD, Garcia-Fontanals A, Blanco-Hinojo L, Garcia-Blanco S, Poca-Dias V, Harrison BJ, Contreras-Rodríguez O, Monfort J, Garcia-Fructuoso F, Deus J. Altered functional magnetic resonance imaging responses to nonpainful sensory stimulation in fibromyalgia patients. Arthritis and Rheumatology 2014;66(11):3200-3209.
[56] Martins IP, Gouveia RG, Parreira E. Kinesiophobia in migraine. J Pain 2006;7(6):445-451.
[57] Meredith PJ, Rappel G, Strong J, Bailey KJ. Sensory sensitivity and strategies for coping with pain. The American Journal of Occupational Therapy 2015;69(4):6904240010p6904240011-6904240010p6904240010.
[58] Midenfjord I, Grinsvall C, Koj P, Carnerup I, Törnblom H, Simrén M. Central sensitization and severity of gastrointestinal symptoms in irritable bowel syndrome, chronic pain syndromes, and inflammatory bowel disease. Neurogastroenterology and Motility 2021.
[59] Naliboff BD, Locke K, Jr., Schrepf AD, Griffith JW, Moldwin R, Krieger JN, Rodriguez LV, Clemens JQ, Lai HH, Sutcliffe S, Taple BJ, Williams D, Pontari MA, Mullins C, Landis JR. Reliability and Validity of Pain and Urinary Symptom Severity Assessment in Urological Chronic Pelvic Pain: A MAPP Network Analysis. J Urol 2022;207(6):1246-1255.
[60] Ogrodniczuk JS, Kealy D, Joyce AS, Abbass AA. Body talk: Sex differences in the influence of alexithymia on physical complaints among psychiatric outpatients. Psychiatry Res 2018;261:168-172.
[61] Özsoy F, Okan S. Somatosensory Amplification, Health Anxiety and Pain Catastrophizing in Individuals with Chronic Musculoskeletal System Pain. Erciyes Medical Journal/Erciyes Tip Dergisi 2021;43(3).
[62] Park SP, Seo JG, Lee WK. Osmophobia and allodynia are critical factors for suicidality in patients with migraine. Journal of Headache and Pain 2015;16(1).
[63] Pearl TA, Dumkrieger G, Chong CD, Dodick DW, Schwedt TJ. Sensory Hypersensitivity Symptoms in Migraine With vs Without Aura: Results From the American Registry for Migraine Research. Headache 2020;60(3):506-514.
[64] Perkins M, de Bruyne M, Giummarra MJ. A pain in the bud? Implications of cross-modal sensitivity for pain experience. Atten Percept Psychophys 2016;78(8):2348-2356.
[65] Peters ML, Vlaeyen JWS, van Drunen C. Do fibromyalgia patients display hypervigilance for innocuous somatosensory stimuli? Application of a body scanning reaction time paradigm. Pain 2000;86(3):283-292.
[66] Pierce J, Hassett AL, Brummett CM, McAfee J, Sieberg C, Schrepf A, Harte SE. Characterizing Pain and Generalized Sensory Sensitivity According to Trauma History Among Patients With Knee Osteoarthritis. Ann Behav Med 2021;55(9):853-869.
[67] Powell G, Derry-Sumner H, Shelton K, Rushton S, Hedge C, Rajenderkumar D, Sumner P. Visually-induced dizziness is associated with sensitivity and avoidance across all senses. J Neurol 2020;267(8):2260-2271.
[68] Price A, Sumner P, Powell G. Subjective sensory sensitivity and its relationship with anxiety in people with probable migraine. Headache 2021;61(9):1342-1350.

[69] Rao EM, Lawrence MM, Hayek SM, Klatzky RL, Carroll BT. Assessing sensory hypersensitivity in interventional pain patients: a pilot study. Regional anesthesia and pain medicine 2023.
[70] Raphael KG, Marbach JJ, Gallagher RM. Somatosensory amplification and affective inhibition are elevated in myofascial face pain. Pain Medicine 2000;1(3):247-253.
[71] Rost S, Van Ryckeghem DM, Schulz A, Crombez G, Vögele C. Generalized hypervigilance in fibromyalgia: Normal interoceptive accuracy, but reduced self-regulatory capacity. J Psychosom Res 2017;93:48-54.
[72] Ryu J, Bar-Shalita T, Granovsky Y, Weissman-Fogel I, Torres EB. Personalized biometrics of physical pain agree with psychophysics by participants with sensory over responsivity. Journal of Personalized Medicine 2021;11(2):1-17.
[73] Sayar K, Barsky AJ, Gulec H. Does somatosensory amplification decrease with antidepressant treatment? Psychosomatics: Journal of Consultation and Liaison Psychiatry 2005;46(4):340-344.
[74] Schrepf A, Williams DA, Gallop R, Naliboff BD, Basu N, Kaplan C, Harper DE, Landis JR, Clemens JQ, Strachan E, Griffith JW, Afari N, Hassett A, Pontari MA, Clauw DJ, Harte SE. Sensory sensitivity and symptom severity represent unique dimensions of chronic pain: a MAPP Research Network study. Pain 2018;159(10):2002-2011.
[75] Schrepf A, Hellman KM, Bohnert AM, Williams DA, Tu FF. Generalized sensory sensitivity is associated with comorbid pain symptoms: A replication study in women with dysmenorrhea. Pain 2023;164(1):142-148.
[76] Schroeder S, Achenbach S, Körber S, Nowy K, de Zwaan M, Martin A. Cognitive-perceptual factors in noncardiac chest pain and cardiac chest pain. Psychosomatic Medicine 2012;74(8):861-868.
[77] Schroeder S, Gerlach AL, Martin A. Implicit affective evaluation of somatosensory sensations in patients with noncardiac chest pain. J Behav Ther Exp Psychiatry 2014;45(3):381-388.
[78] Seo JG, Park SP. Clinical significance of sensory hypersensitivities in migraine patients: does allodynia have a priority on it? Neurol Sci 2019;40(2):393-398.
[79] Sinclair C, Meredith P, Strong J, Chalkiadis GA. Sensory Modulation: An Important Piece of the Disability Puzzle for Adolescents With Persistent Pain. Clin J Pain 2019;35(2):121-132.
[80] Sinclair C, Meredith P, Strong J. Pediatric Persistent Pain: Associations Among Sensory Modulation, Attachment, Functional Disability, and Quality of Life. American Journal of Occupational Therapy 2020;74(2):1-11.
[81] Stewart DE, Reicher AE, Gerulath AH, Boydell KM. Vulvodynia and psychological distress. Obstetrics and Gynecology 1994;84(4 I):587-590.
[82] Subaşi Tekintaş N, Yanartaş Ö, Benk Durmuş F, Sayar K. Functional Somatic Symptoms and Their Predictors in Patients with Major Depressive Disorder and Fibromyalgia Syndrome. Noropsikiyatri Arsivi 2022;59(4):274-280.
[83] Sullivan JC, Miller LJ, Nielsen DM, Schoen SA. The presence of migraines and its association with sensory hyperreactivity and anxiety symptomatology in children with autism spectrum disorder. Autism 2014;18(6):743-747.
[84] Suzuki K, Suzuki S, Shiina T, Okamura M, Haruyama Y, Tatsumoto M, Hirata K. Investigating the relationships between the burden of multiple sensory hypersensitivity symptoms and headache-related disability in patents with migraine. Journal of Headache & Pain 2021;22(1):1-6.
[85] Ten Brink AF, Peters L, Kompouli PI, Jordan A, McCabe CS, Goebel A, Bultitude JH. Bodily changes and sensory sensitivity in complex regional pain syndrome and fibromyalgia. Pain 2020;161(6):1361-1370.
[86] Vrbanović E, Zlendić M, Alajbeg IZ. Association of oral behaviours' frequency with psychological profile, somatosensory amplification, presence of pain and self-reported pain intensity. Acta odontologica Scandinavica 2022;80(7):522-528.

[87] Wang D, Merkle SL, Lee JE, Sluka KA, Rakel B, Graven-Nielsen T, Frey-Law LA. Multisensory Sensitivity is Related to Deep-Tissue but Not Cutaneous Pain Sensitivity in Healthy Individuals. J Pain Res 2020;13:2493-2508.
[88] Wang D, Casares S, Eilers K, Hitchcock S, Iverson R, Lahn E, Loux M, Schnetzer C, Frey-Law LA. Assessing Multisensory Sensitivity Across Scales: Using the Resulting Core Factors to Create the Multisensory Amplification Scale. J Pain 2022;23(2):276-288.
[89] Wang D, Frey-Law LA. Multisensory sensitivity differentiates between multiple chronic pain conditions and pain-free individuals. Pain 2023;164(2):E91-E102.
[90] Weissman-Fogel I, Granovsky Y, Bar-Shalita T. Sensory Over-Responsiveness among Healthy Subjects is Associated with a Pronociceptive State. Pain Pract 2018;18(4):473-486.
[91] Wilbarger JL, Cook DB. Multisensory hypersensitivity in women with fibromyalgia: Implications for well being and intervention. Archives of Physical Medicine and Rehabilitation 2011;92(4):653-656.
[92] Yavuz BG, Aydinlar EI, Dikmen PY, Incesu C. Association between somatic amplification, anxiety, depression, stress and migraine. J Headache Pain 2013;14(1):53.

Appendix A: PubMed Search String: *as searched 11/8/21 – 5,457 Results*
((((“multisensory”[tiab] OR
“sensory”[tiab] OR
“somatosensory”[tiab] OR
“generalized sensory”[tiab]
) AND
(“sensitivity”[tiab] OR
“altered processing”[tiab] OR
“avoidance”[tiab] OR
“hypersensitivity”[tiab] OR
“hypersensitivities”[tiab] OR
“amplification”[tiab] OR
“defensiveness”[tiab] OR
“hypervigilance”[tiab] OR
“overresponsiveness”[tiab] OR
“over-responsiveness”[tiab] OR
“overresponsivity”[tiab] OR
“over-responsivity”[tiab] OR
“overresponsivities”[tiab] OR
“over-responsivities”[tiab] OR
“defensivity”[tiab] OR
“reactivity”[tiab] OR
“modulation”[tiab] OR
“hyperreactivity”[tiab] OR
“hyper-reactivity”[tiab]
)) OR
((Adult[tiab] AND Sensory[tiab] AND Profile[tiab]) OR
(Highly[tiab] AND Sensitive[tiab] AND Person[tiab]) OR
(Glasgow[tiab] AND Sensory[tiab] AND Questionnaire[tiab]) OR (Sensory[tiab AND Over-Responsivity[tiab] AND Scale[tiab]) OR
(Sensory[tiab] AND Processing[tiab] AND Questionnaire[tiab]) OR (Sensory[tiab] AND Sensitivity[tiab] AND Scales[tiab]) OR
(Somatosensory[tiab] AND Amplification[tiab] AND Scale[tiab]) OR
(Generalized[tiab] AND Sensory[tiab] AND Sensitivity[tiab]) OR
(Short[tiab] AND Sensory[tiab] AND Profile[tiab]) OR
(Sensory[tiab] AND Integration[tiab] AND Inventory[tiab]) OR
(Sensory[tiab] AND Hypersensitivity[tiab] AND Scale[tiab]) OR
(Multisensory[tiab] AND Amplification[tiab] AND Scale[tiab]) OR
(Pain[tiab] AND Sensitivity[tiab] AND Questionnaire[tiab])
)) AND
(“chronic pain”[MeSH] OR
“pain measurement”[MeSH] OR
“fibromyalgia”[MeSH] OR
“hyperesthesia”[MeSH] OR

"migraine disorders"[Mesh:NoExp] OR
"headache disorders, primary"[MeSH:NoExp] OR
"arthritis"[Mesh:NoExp] OR
"irritable bowel syndrome"[MeSH] OR
"carpal tunnel syndrome"[MeSH] OR
"tendinopathy"[MeSH:NoExp] OR
"temporomandibular joint disorders"[MeSH] OR
"fasciitis, plantar"[MeSH] OR
"headache"[Mesh] OR
"Complex Regional Pain Syndromes"[Mesh] OR
"vulvodynia"[MeSH] OR
"neuralgia"[Mesh:NoExp] OR
"nociceptive pain"[MeSH] OR
"myalgia"[MeSH] OR
"hyperalgesia"[MeSH] OR
"central nervous system sensitization"[Mesh:NoExp] OR
"chronic pain"[tiab] OR
"physical suffering"[tiab] OR
"ache"[tiab] OR
"arthralgia"[tiab] OR
"aches"[tiab] OR
"hyperesthesia"[tiab] OR
"postsynaptic summation"[tiab] OR
"postsynaptic potential summation"[tiab] OR
"wind up"[tiab] OR
"fibromyalgia"[tiab] OR
"muscular rheumatism"[tiab] OR
"fibrositis"[tiab] OR
"migraine"[tiab] OR
"migraine disorders"[tiab] OR
"status migrainosus"[tiab] OR
"sick headache"[tiab] OR
"arthritis"[tiab] OR
"arthritides"[tiab] OR
"irritable bowel syndrome"[tiab] OR
"irritable colon"[tiab] OR
"mucous colitis"[tiab] OR
"mucous colitides"[tiab] OR
"myalgic encephalomyelitis"[tiab] OR
"royal free disease"[tiab] OR
"chronic fatigue disorder"[tiab] OR
"systemic exertion intolerance disease"[tiab] OR
"carpal tunnel syndrome"[tiab] OR
"CRPS"[tiab] OR

"whiplash"[tiab] OR
"tendonitis"[tiab] OR
"tendonitides"[tiab] OR
"tendinitis"[tiab] OR
"tendonosis"[tiab] OR
"tendinoses"[tiab] OR
"tendinosis"[tiab] OR
"temporomandibular disorder"[tiab] OR
"temporomandibular joint disorder"[tiab] OR
"temporomandibular joint disorders"[tiab] OR
"TMJ disorders"[tiab] OR
"temporomandibular disorders"[tiab] OR
"temporomandibular joint diseases"[tiab] OR
"TMJ diseases"[tiab] OR
"Plantar fasciitis"[tiab] OR
"fasciitis, plantar"[tiab] OR
"Policeman's heel"[tiab] OR
"heel spur syndrome"[tiab] OR
"headache"[tiab] OR
"cephalodynia"[tiab] OR
"cephalalgia"[tiab] OR
"hemicrania"[tiab] OR
"Reflex Sympathetic Dystrophy"[tiab] OR
"CRPS Type I"[tiab] OR
"Reflex sympathetic dystrophy syndrome"[tiab] OR
"shoulder-hand syndrome"[tiab] OR
"algodystrophy"[tiab] OR
"vulvodynia"[tiab] OR
"vestibulodynia"[tiab] OR
"neuralgia"[tiab] OR
"neurodynia"[tiab] OR
"nociceptive pain"[tiab] OR
"central sensitization"[tiab] OR
"central nervous system sensitization"[tiab] OR
"hyperalgesia"[tiab] OR
"hyperalgia"[tiab] OR
"allodynia"[tiab] OR
"myalgia"[tiab] OR
"muscle soreness"[tiab] OR
"muscle tenderness"[tiab] OR
"central pain"[tiab]
))
NOT ("Animals"[Mesh] NOT ("Animals"[Mesh] AND "Humans"[Mesh]))
